# The liver-specific long noncoding RNA FAM99B inhibits ribosome biogenesis and cancer progression through cleavage of dead-box Helicase 21

**DOI:** 10.1038/s41419-025-07401-w

**Published:** 2025-02-14

**Authors:** Yifei He, Hongquan Li, Qili Shi, Yanfang Liu, Qiaochu Pan, Xianghuo He

**Affiliations:** 1 Fudan University Shanghai Cancer Center and Institutes of Biomedical Sciences; Department of Oncology, Shanghai Medical College, Fudan University, Shanghai, 200032 China; 2 Key Laboratory of Breast Cancer in Shanghai, Fudan University Shanghai Cancer Center, Fudan University, Shanghai, 200032 China

**Keywords:** Targeted therapies, Mechanisms of disease

## Abstract

Emerging evidence has demonstrated that long noncoding RNAs (lncRNAs) are promising targets or agents for the treatment of human cancers. Most liver-specific lncRNAs exhibit loss of expression and act as tumor suppressors in liver cancer. Modulating the expression of these liver-specific lncRNAs is a potential approach for lncRNA-based gene therapy for hepatocellular carcinoma (HCC). Here, we report that the expression of the liver-specific lncRNA FAM99B is significantly decreased in HCC tissues and that FAM99B suppresses HCC cell proliferation and metastasis both in vitro and in vivo. FAM99B promotes the nuclear export of DDX21 through XPO1, leading to further cleavage of DDX21 by caspase3/6 in the cytoplasm. FAM99B inhibits ribosome biogenesis by inhibiting ribosomal RNA (rRNA) processing and RPS29/RPL38 transcription, thereby reducing global protein synthesis through downregulation of DDX21 in HCC cells. Interestingly, the FAM99B^65-146^ truncation exhibits tumor-suppressive effects in vivo and in vitro. Moreover, GalNAc-conjugated FAM99B^65-146^ inhibits the growth and metastasis of orthotopic HCC xenografts, providing a new strategy for the treatment of HCC. This is the first report of the use of a lncRNA as an agent rather than a target in tumor treatment.

**Figure:**
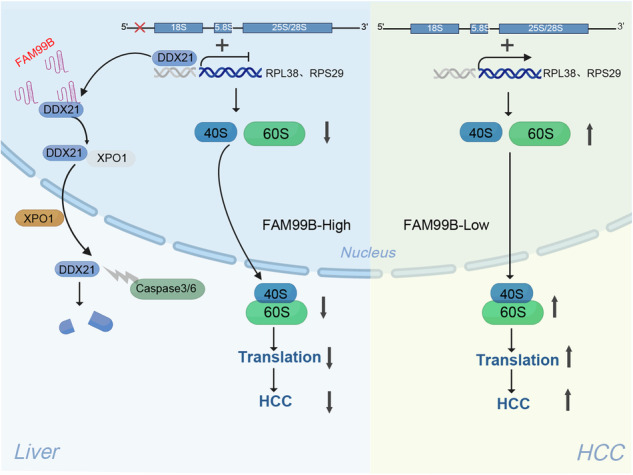
Graphical illustration of the mechanism of FAM99B in HCC.

Graphical illustration of the mechanism of FAM99B in HCC.

## Introduction

Long noncoding RNA (lncRNA) play crucial regulatory roles in tumors and are increasingly being applied in cancer therapy. LncRNAs regulate tumor cell proliferation, invasion, metastasis, and chemoresistance by influencing chromosome silencing, genomic imprinting, chromatin remodeling, transcriptional regulation, and nuclear transport [1], thereby impacting tumor initiation and progression [2]. Emerging evidence indicates that lncRNAs are promising targets or therapeutic agents for cancer treatment. A variety of RNA-based therapeutic agents and strategies have been developed, including antisense oligonucleotides (ASOs), small interfering RNAs (siRNAs), short hairpin RNAs (shRNAs), miRNA mimics, therapeutic circular RNAs (circRNAs), and CRISPR/Cas9-based gene editing [3–5].

Liver cancer is a significant global health threat. Primary liver cancer is the sixth most common cancer worldwide and is the second-leading cause of cancer-related death in men and the sixth-leading cause of cancer death in women [6]. The 5-year survival rate of patients with hepatocellular carcinoma (HCC) is still only ~20% [7]. The poor prognosis of HCC is attributed to its early asymptomatic nature, which lead to late diagnosis, and its significant resistance to traditional chemotherapy and radiotherapy [8]. Studying the mechanisms of HCC occurrence and development, identifying new tumor markers, and developing novel therapeutic approaches to extend the survival period of patients are urgent priorities. Tissue-specific lncRNAs often perform specific functions in tissues. Notably, most liver-specific lncRNAs exhibit loss of expression in liver cancer, and usually suppress tumor development and progression [9–12]. Therefore, regulating the expression of these liver-specific lncRNAs is a potential strategy for lncRNA-based gene therapy for HCC.

Studies have reported that FAM99B in exosomes derived from human umbilical cord mesenchymal stem cells (hucMSC-Exo) induces cell cycle arrest and apoptosis, suppressing HCC development and progression [13]. Additionally, researchers have predicted that FAM99B is involved in “metabolic pathways” and “blood coagulation” pathways [11]. Other studies have indicated that FAM99B is associated with the response to wounding, lipid biosynthesis, and the regulation of lipid metabolism [12]. However, the molecular mechanisms of FAM99B in tumors remain unexplored and lack experimental validation.

In our study, FAM99B exerts its function through interaction with DDX21. DDX21 (Dead-Box Helicase 21), a member of the DEAD-box family, is an RNA helicase that uses the energy generated by ATP hydrolysis to catalyze the unwinding of double-stranded RNA or RNA‒DNA hybrids [14]. DDX21 is recognized as an important nucleolar protein involved in ribosomal RNA (rRNA) processing and plays a multifaceted role in multiple steps of ribosome biogenesis [15]. Previous studies have shown that depletion of DDX21 results in significant reductions in the 18S and 28S rRNA levels in numerous cell types [16, 17]. DDX21 contributes to the development of multiple human cancers, including breast cancer [18], gastric cancer [19], melanoma [20], colorectal cancer [21], and neuroblastoma [22], but its role in HCC has not been reported.

In this study, we report a liver-specific lncRNA, FAM99B, which is strongly downregulated in HCC and serves as an independent predictor of overall survival in HCC patients. FAM99B interacts with DDX21 and decreases the DDX21 protein level via caspase3/6-mediated cleavage in HCC cells. FAM99B inhibits ribosome biogenesis and protein synthesis by downregulating DDX21, thus suppressing the proliferation and metastasis of HCC cells. Notably, the FAM99B^65-146^ truncation can significantly inhibit the progression of HCC, and N-acetylgalactosamine (GalNAc)-conjugated FAM99B^65-146^ (GalNAc-FAM99B^65-146^) attenuates the growth and metastasis of orthotopic tumor xenografts in vivo, providing a new strategy and approach for the treatment of HCC.

## Results

### The liver-specific lncRNA FAM99B acts as a tumor suppressor in HCC

**Fig. 1:**
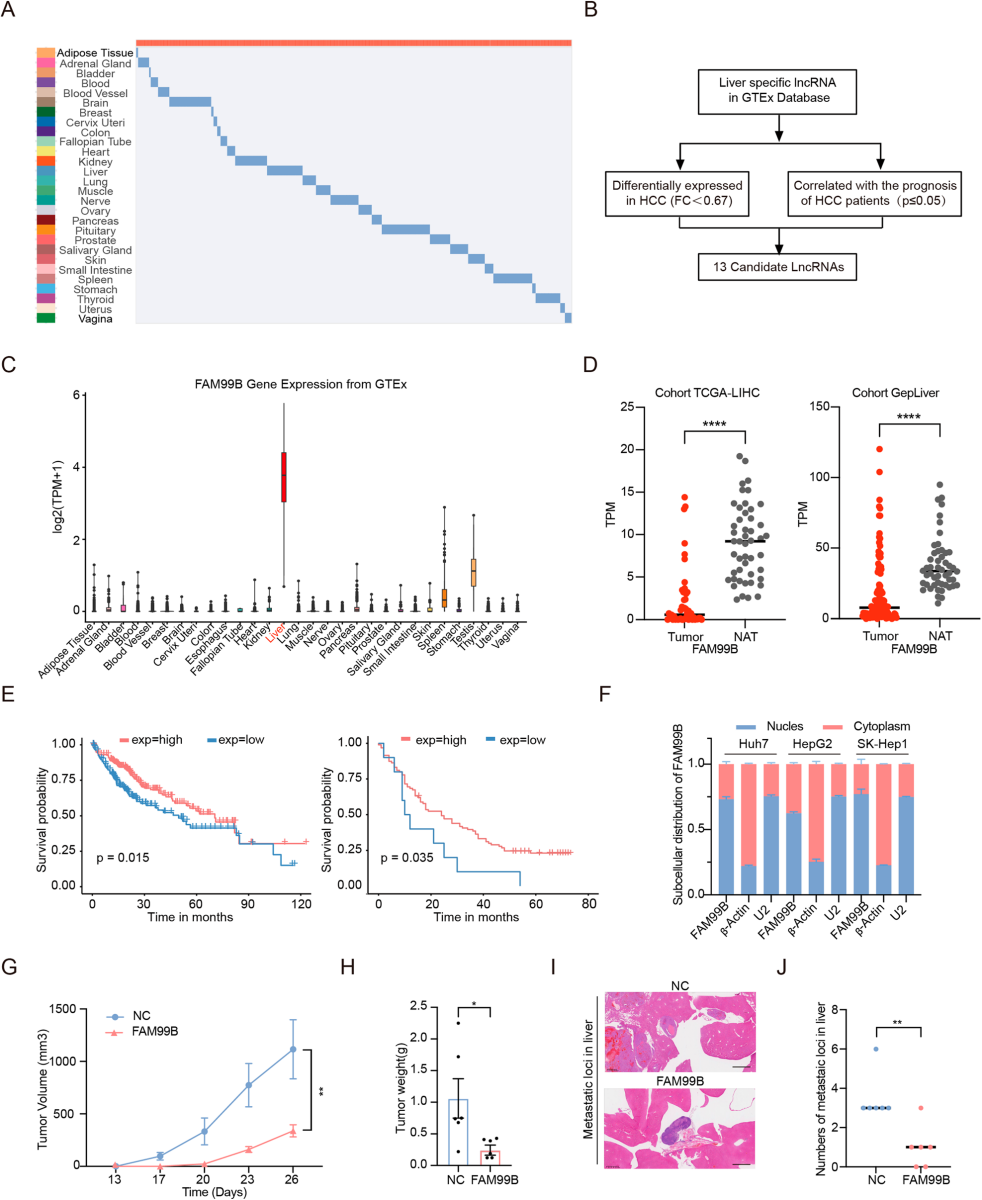
The liver-specific lncRNA FAM99B is downregulated in HCC and inhibits the proliferation and metastasis of HCC cells both and in vivo. **A** Tissue-specific lncRNAs in the GTEx database. **B** Flowchart of the screening procedure for liver-specific lncRNAs. **C** Expression of FAM99B in different tissues. **D** Relative expression of FAM99B in HCC tissues and normal tumor-adjacent tissues (NATs) in the TCGA-LIHC cohort (left) and the GepLiver project cohort (right). Data are represented as the mean ± SD (*n* = 2). Unpaired *t*-test. **E** Kaplan–Meier analysis of overall survival in HCC patients in the TCGA-LIHC cohort (left) and the GepLiver project cohort (right). **F** Subcellular localization of FAM99B as determined by nuclear‒cytoplasmic fractionation in Huh7, HepG2 and SK-Hep1 cells. Data are represented as the mean ± SD (*n* = 3). **G**, **H** Tumor volumes (**G**) and tumor weights (**H**) in subcutaneous tumor-bearing mice in the FAM99B overexpression group and control group. Data are represented as the mean ± SD (*n* = 3). Unpaired *t*-test. **I** Representative images of H&E-stained liver metastases from nude mice injected with Huh7 cells. **J** Numbers of liver metastases in the control group and FAM99B overexpression group. Data are represented as the mean ± SD (*n* = 3). Unpaired *t*-test. *p < 0.05, **p < 0.01, ***p < 0.001, ****p < 0.0001, and ns, not significant.

First, we systematically analyzed tissue-associated RNA-seq data in the Genotype-Tissue Expression (GTEx) database and identified numerous tissue-specific lncRNAs expressed in different human tissues (Supplemental Table S1, Fig. 1A). Furthermore, we screened for liver-specific lncRNAs by using the Liver Hepatocellular Carcinoma (LIHC) dataset from The Cancer Genome Atlas (TCGA; https://portal.gdc.cancer.gov/) and identified 13 candidates that exhibited downregulated expression (fold change <0.67) in liver cancer patients and were associated with a better prognosis in HCC (*p* ≤ 0.05) (Supplemental Table S1, Fig. 1B). Notably, FAM99B was expressed almost exclusively in the liver (Fig. 1C). Moreover, we analyzed data from 370 HCC patients in the TCGA-LIHC cohort and 105 HCC patients in the GepLiver project cohort (http://www.gepliver.org/). The results revealed that FAM99B was downregulated in HCC (Fig. 1D), and a similar result was found in two different HCC datasets in the Gene Expression Omnibus (GEO) database (GSE77314 and GSE144269) (Supplemental Fig. S1A). Further analysis revealed that high expression of FAM99B was associated with a better prognosis in HCC (Fig. 1E and Supplemental Fig. S1B). The full-length sequence of FAM99B contains 1066 nucleotides, as identified by 3’ rapid amplification of cDNA ends (RACE) and 5’ RACE (Supplemental Fig. S1C and S1D). Analysis of subcellular localization through nuclear‒cytoplasmic fractionation and immunofluorescence staining revealed that FAM99B was localized predominantly in the nucleus in hepatocytes (Fig. 1F and Supplemental Fig. S1E).

Cell Counting Kit-8 (CCK-8) and colony formation assays revealed that the overexpression of FAM99B significantly inhibited the proliferation of HCC cells (Supplemental Fig. S2A–D). Migration and invasion assays revealed that FAM99B significantly inhibited the migration and invasion of HCC cells (Supplemental Fig. S2E). To further validate the biological function of FAM99B in vivo, overexpressed FAM99B and control Huh7 cells were subcutaneously injected into the flanks of nude mice, and body weight changes and tumor formation were monitored regularly. The results revealed that FAM99B-overexpressing tumors grew much slower (Fig. 1G and Supplemental Fig. S2F) and weighed less (Fig. 1H) than control tumors. In addition, Ki-67 staining revealed that the cell proliferation rate in tumors formed from FAM99B-overexpressing cells was lower than that in tumors formed from control cells (Supplemental Fig. S2G and S2H). Next, we established an orthotopic xenograft mouse model via intrahepatic injection of Huh7 cells with stable overexpression of FAM99B to evaluate the effects of FAM99B on HCC metastasis. In this model, overexpression of FAM99B dramatically inhibited HCC cell metastasis (Supplemental Fig. S2I and S2J, Fig. 1I and J). These results indicate that FAM99B is a bona fide tumor suppressor in HCC.

### FAM99B exerts its biological functions through an interaction with DDX21

**Fig. 2:**
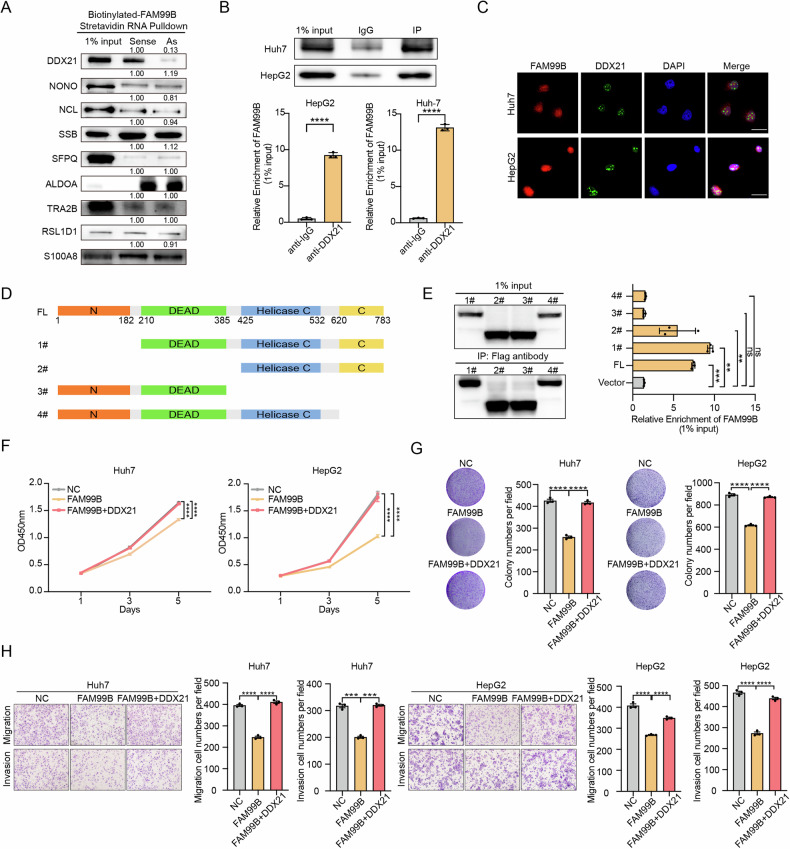
FAM99B exerts its biological functions through an interaction with DDX21 in HCC cells. **A** Western blot analysis was performed to validate the candidates identified via FAM99B RNA pulldown coupled with MS. **B** RNA immunoprecipitation was performed in Huh7 and HepG2 cells with an anti-DDX21 antibody, and the enrichment of FAM99B was evaluated by qPCR. **C** The colocalization of FAM99B and DDX21 in Huh7 and HepG2 cells was evaluated by immunofluorescence staining. Scale bar, 25 μm. **D**, **E** Schematic diagram of the DDX21 truncations (**D**) and the binding of the DDX21 truncations to FAM99B, as evaluated via RIP assays in cells transiently transfected with the Flag-tagged full-length (FL) DDX21 and DDX21 truncation constructs. Data are represented as the mean ± SD (*n* = 3). One-way ANOVA with correction for multiple comparisons (**E**). **F**–**H** CCK-8 assays (**F**), colony formation assays (**G**), and migration and invasion assays (**H**) of Huh7 and HepG2 cells following overexpression of FAM99B and DDX21. Data are represented as the mean ± SD (n = 3). Two-way ANOVA with correction for multiple comparisons (**F**) and (**H**). One-way ANOVA with correction for multiple comparisons (**G**). *p < 0.05, **p < 0.01, ***p < 0.001, ****p < 0.0001, and ns, not significant.

To explore the molecular mechanisms by which FAM99B suppresses the proliferation and metastasis of HCC cells, we performed an RNA pulldown assay coupled with mass spectrometry (MS) to screen for potential binding partners of FAM99B. In our analysis of the results of MS analysis of the RNA pulldown products, we screened for proteins with a coverage (the percentage of the protein sequence covered by identified peptides) of ≥5 and a fold change between sense/antisense transcripts of ≥5. Considering that FAM99B is located in the nucleus, we identified 9 candidates that might interact with FAM99B. Among these candidates, DDX21 was the protein with the most significant binding to FAM99B (Supplemental Fig. S3A, Fig. 2A and B). Immunofluorescence staining revealed colocalization of FAM99B and DDX21 in the nucleus in HCC cells (Fig. 2C). To clarify which domain of DDX21 binds to FAM99B, we next constructed Flag-tagged full-length DDX21 and truncation mutants of DDX21 on the basis of its functional domains (fragment #1: 210–783 aa, fragment #2: 425–783 aa, fragment #3: 1–385 aa, and fragment 4: 1–620 aa) (Fig. 2D). In the subsequent RNA immunoprecipitation (RIP) assays, we found that deletion of the C-terminal domain dramatically impaired the FAM99B–DDX21 interaction (Fig. 2E). These results indicate that FAM99B binds to the C-terminal domain of DDX21.

Previous reports have shown that DDX21 acts as a tumor promoter in several cancers [19–22]. However, the function of DDX21 in HCC is still unclear. Our results demonstrated that overexpression of DDX21 promoted the growth, colony formation, migration and invasion of HCC cells (Supplemental Fig. S3B–D and S3G). However, knockdown of DDX21 inhibited these behaviors of HCC cells (Supplemental Fig. S3E, S3F and S3H). To determine whether DDX21 is a functional effector of FAM99B, we overexpressed FAM99B and DDX21 in Huh7 and HepG2 cells. The results revealed that overexpression of DDX21 abolished the inhibitory effects of FAM99B on HCC cells (Supplemental Fig. S3I and Fig. 2F, G, Fig. 2H**)**. These results suggest that FAM99B reduces the proliferation and metastasis of HCC cells by downregulating DDX21.

### FAM99B promotes the nuclear export of DDX21 and its cleavage by Casp3/6

**Fig. 3:**
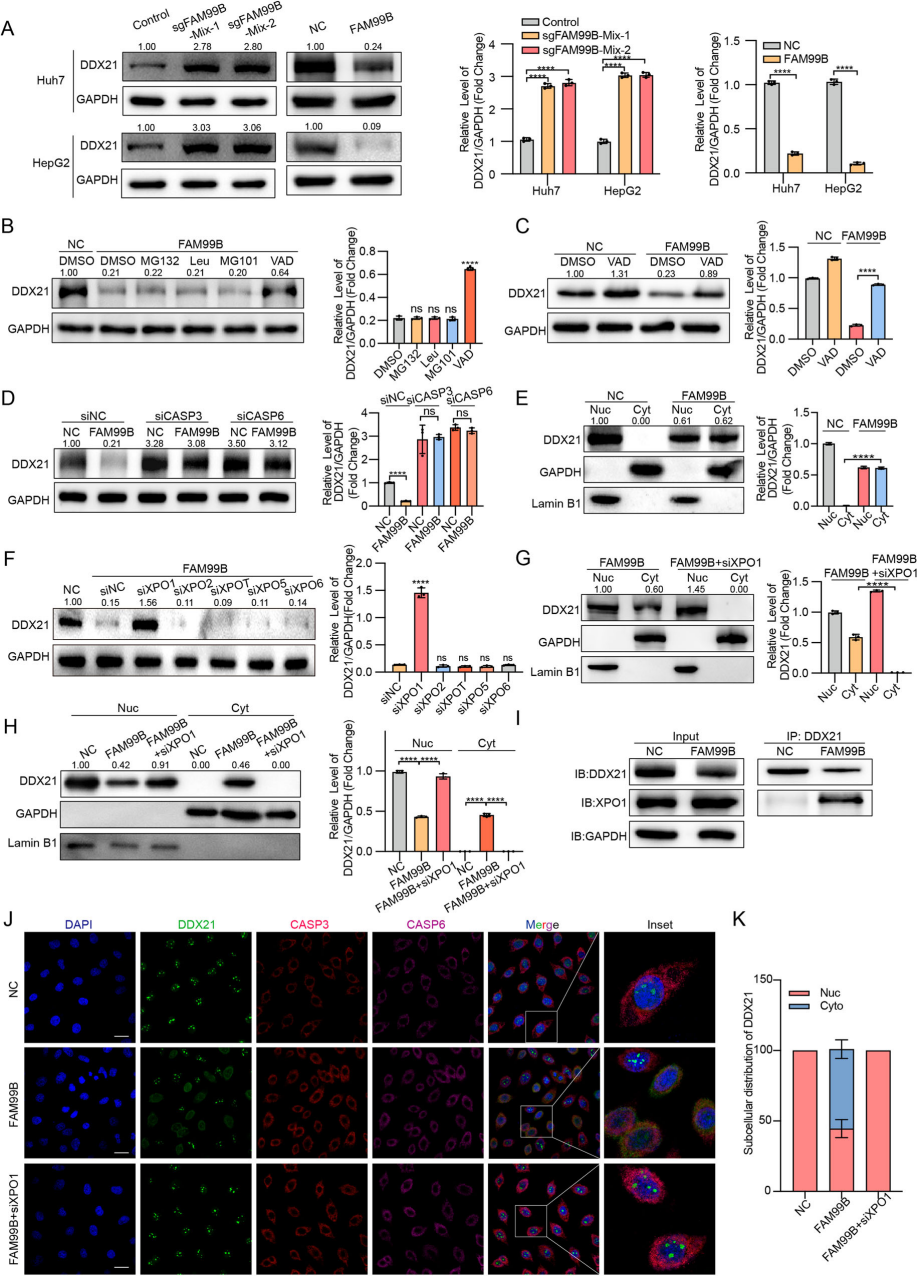
FAM99B promotes the nuclear export of DDX21 and its cleavage by casp3/6. **A** Western blot analysis of the DDX21 protein level in Huh7 and HepG2 cells after overexpression or knockout of FAM99B, followed by statistical analysis. Data are represented as the mean ± SD (*n* = 3). Unpaired *t*-test or One-way ANOVA with correction for multiple comparisons. **B** Huh7 cells stably overexpressing FAM99B were treated with dimethyl sulfoxide (DMSO) as the control, 10 μM MG132, 10 μM leupeptin (Leu), 20 μM MG101, or 10 μM z-VAD-FMK (VAD) for 24 h, and the cell lysates were subjected to western blot with anti-DDX21 and anti-GAPDH antibodies, followed by statistical analysis. Data are represented as the mean ± SD (*n* = 3). One-way ANOVA with correction for multiple comparisons. **C** Huh7 cells stably overexpressing FAM99B were treated with DMSO or 10 μM VAD for 24 h, followed by western blot and statistical analysis. Data are represented as the mean ± SD (*n* = 3). Unpaired *t*-test. **D** The protein expression levels of DDX21 after the knockdown of casp3, casp6 and casp7 in Huh7 cells with stable overexpression of FAM99B were measured via western blot, followed by statistical analysis. Data are represented as the mean ± SD (*n* = 3). Unpaired *t*-test. **E** The subcellular localization of DDX21 was evaluated in FAM99B-overexpressing Huh7 cells via western blot, followed by statistical analysis. Data are represented as the mean ± SD (*n* = 3). Unpaired *t*-test. **F** Western blot analysis of DDX21 protein levels in FAM99B-overexpressing Huh7 cells after knockdown of XPO1, XPO2, XPOT, XPO5, and XPO6, followed by statistical analysis. Data are represented as the mean ± SD (*n* = 3). One-way ANOVA with correction for multiple comparisons. **G**, **H** Western blot analysis of DDX21 subcellular localization in Huh7 cells with FAM99B overexpression and XPO1 knockdown, followed by statistical analysis. Data are represented as the mean ± SD (*n* = 3). Unpaired *t*-test or One-way ANOVA with correction for multiple comparisons. **I** Immunoprecipitation was used to detect the association between XPO1 and DDX21 after FAM99B overexpression in Huh7 cells. **J** Immunofluorescence staining was used to observe the localization of DDX21 after FAM99B overexpression and XPO1 knockdown. **K** Quantification of the relative percentages of cells with nuclear or cytoplasmic DDX21 staining was performed in the immunofluorescence experiment involving FAM99B overexpression and subsequent XPO1 knockdown. Ten images were randomly captured from 60 high-power fields (HPFs) across different regions. *p < 0.05, **p < 0.01, ***p < 0.001, ****p < 0.0001, and ns, not significant. Scale bar, 25 μm.

Next, we sought to determine the consequences of the interaction between FAM99B and DDX21 in HCC cells. Neither overexpression nor knockdown of FAM99B affected the mRNA level of DDX21 (Supplemental Fig. S4A). Modulation of DDX21 expression did not affect the expression of FAM99B (Supplemental Fig. S4B). However, overexpression of FAM99B led to a decrease in the protein level of DDX21, whereas knockdown of FAM99B resulted in an increase in the protein level of DDX21 (Fig. 3A). These results indicate that FAM99B can downregulate the protein levels of DDX21.

To explore the cause of these effects on the DDX21 protein level, we treated Huh7 cells with stable FAM99B overexpression with MG132 (proteasome inhibitor), leupeptin (lysosomal inhibitor), MG101 (calpain inhibitor), and Z-VAD-FMK (pancaspase inhibitor). Treatment with Z-VAD-FMK suppressed the FAM99B-induced downregulation of DDX21 at the translational level **(**Fig. 3B, C**)**. The caspase family contains numerous members. We interfered with all the caspase family members expressed in Huh7 and HepG2 cells (Supplemental Fig. S4C) and found that interfering with the expression of caspase3 and caspase6 in Huh7 cells reversed the downregulation of DDX21 caused by FAM99B overexpression. Similarly, in HepG2 cells, interfering with the expression of caspase3, caspase6, and caspase7 restored the downregulation of DDX21 (Supplemental Fig. S4D). The same effects were observed after interference with the expression of caspase3 and caspase6 in Huh7 and HepG2 cells with stable overexpression of FAM99B (Supplemental Fig. S4E and S4F, Fig. 3D). In addition, DDX21 bound to caspase3 and caspase6 in Huh7 cells (Supplemental Fig. S4G). These results indicate that FAM99B might regulate the DDX21 protein level via casp3/6 in HCC cells.

Since the caspase family is usually localized in the cytoplasm, we hypothesized that FAM99B might regulate the nucleocytoplasmic distribution of DDX21. Using nuclear-cytoplasmic fractionation experiments and immunofluorescence analysis, we found that overexpression of FAM99B led to the translocation of DDX21 from the nucleus to the cytoplasm (Fig. 3E and J). We next sought to investigate the mechanism by which DDX21 is exported to the cytoplasm. The majority of macromolecular (>40 kDa) transport across nuclear pores is mediated by the Karyopherin-β family of nuclear transport receptors [23]. To identify the specific nuclear export protein involved, we individually knocked down XPO1, XPO2, XPO3, XPO5, and XPO6 in FAM99B-overexpressing Huh7 cells, followed by nuclear-cytoplasmic fractionation (Supplemental Fig. S4H). The results revealed that knockdown of XPO1 effectively inhibited FAM99B-induced nuclear export of DDX21, whereas silencing other exportins did not affect DDX21 localization (Supplemental Fig. S4I). This suggests that XPO1 may be the exportin mediating the nuclear export of DDX21 in FAM99B-overexpressing cells. To further validate this finding, we performed XPO1 knockdown experiments in FAM99B-overexpressing HepG2 cells, followed by nuclear-cytoplasmic fractionation and immunofluorescence analysis. XPO1 depletion significantly reduced the cytoplasmic distribution of DDX21, almost abolishing it, while concurrently increasing its nuclear localization (Fig. 3F and H, Supplemental Fig. S4H). Moreover, co-immunoprecipitation assays demonstrated that FAM99B overexpression enhanced the interaction between DDX21 and XPO1. Collectively, these results indicate that in HCC cells, FAM99B overexpression recruits the export protein XPO1 to facilitate the translocation of DDX21 from the nucleus to the cytoplasm.

DDX21 was reported to be cleaved at D126 by casp3/6 in response to vesicular stomatitis virus infection in HeLa cells [24]. Thus, we transfected plasmids containing Flag-tagged DDX21^WT^ and the DDX21^D126A^ mutant into Huh7 cells and found that the DDX21^WT^ protein level was significantly reduced but the DDX21^D126A^ protein level was not decreased (Supplemental Fig. S4J). These results indicate that FAM99B promotes the export and degradation of DDX21 via casp3/6-mediated cleavage at D126.

### FAM99B inhibits mRNA translation through suppression of DDX21 expression in HCC cells

**Fig. 4:**
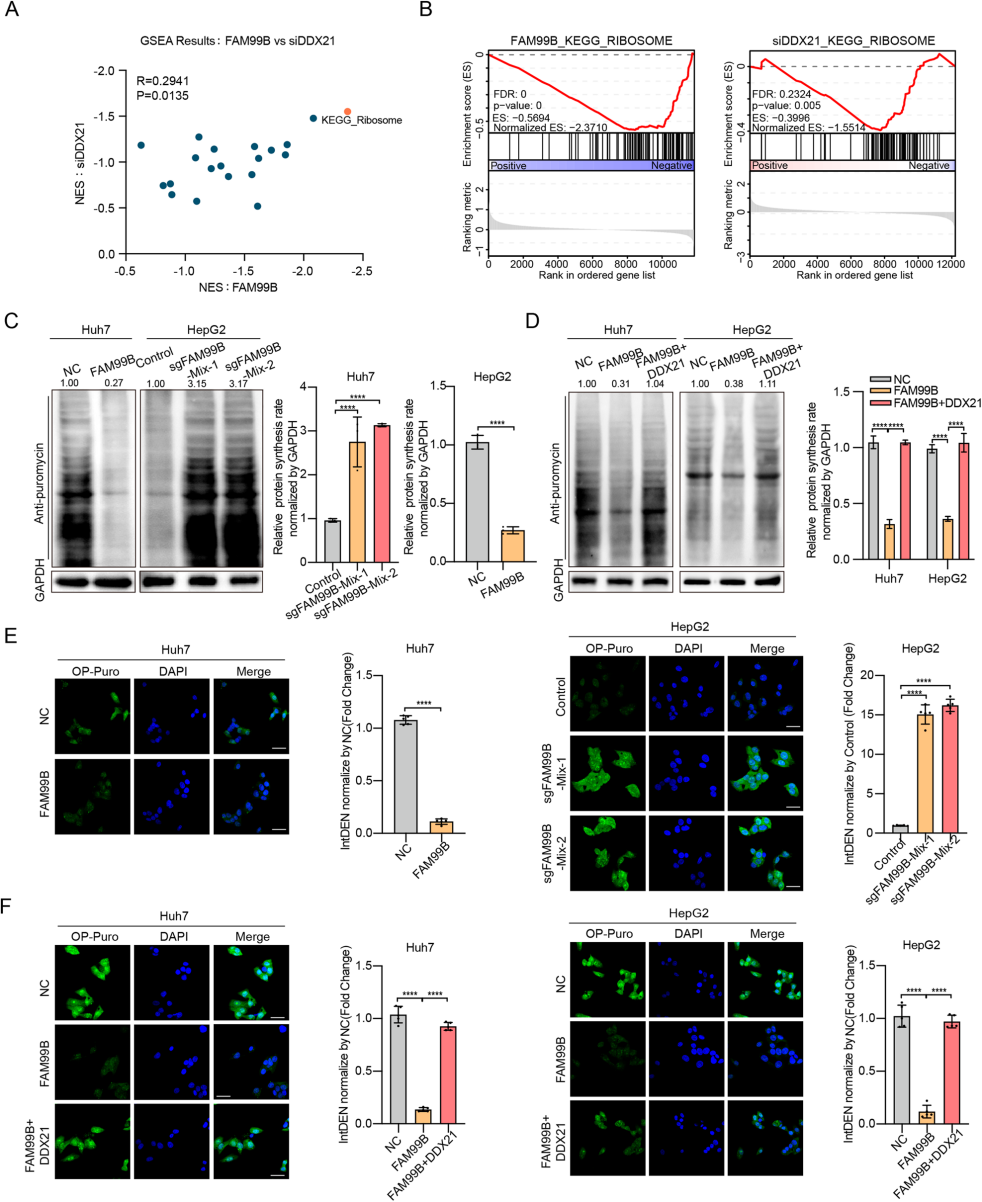
FAM99B inhibits mRNA translation through suppression of DDX21 expression in HCC cells. **A** GSEA was performed in FAM99B-overexpressing and DDX21-knockdown Huh7 cells. The horizontal axis shows the fold enrichment score for each signaling pathway, and the vertical axis shows the fold enrichment score for each signaling pathway after DDX21 was knocked down with siRNA. **B** GSEA of the ribosome pathway in Huh7 cells with FAM99B overexpression (left) and DDX21 knockdown (right). **C** Huh7 cells overexpressing FAM99B and HepG2 cells with FAM99B knockout were treated with 10 μg/ml puromycin for 30 min, followed by Western blot and statistical analysis. Data are represented as the mean ± SD (*n* = 3). Unpaired *t*-test or One-way ANOVA with correction for multiple comparisons. **D** Huh7 and HepG2 cells co-expressing FAM99B and DDX21 were treated with 10 μg/ml puromycin for 30 min, followed by Western blot and statistical analysis. Data are represented as the mean ± SD (*n* = 3). One-way ANOVA with correction for multiple comparisons. **E** The effects of FAM99B overexpression (left) and knockout (right) on protein synthesis in Huh7 and HepG2 cells were evaluated via an OP-Puro assay. Randomly select 5 fields of view, calculate the fluorescence Integrated Density (IntDen) using ImageJ, and perform statistical analysis. Data are represented as the mean ± SD (*n* = 5). Unpaired *t-*test or One-way ANOVA with correction for multiple comparisons. **F** The effects of FAM99B and DDX21 overexpression on protein synthesis in Huh7 and HepG2 cells were evaluated via an OP-Puro assay. Randomly select 5 fields of view, calculate the fluorescence Integrated Density (IntDen) using ImageJ, and perform statistical analysis. Data are represented as the mean ± SD (*n* = 5). One-way ANOVA with correction for multiple comparisons. *p < 0.05, **p < 0.01, ***p < 0.001, ****p < 0.0001, and ns, not significant. Scale bar, 25 μm.

To explore the molecular pathway of FAM99B and DDX21 in HCC cells, we performed RNA-seq after overexpression of FAM99B or knockdown of DDX21. The gene set enrichment analysis (GSEA) results revealed that genes related to the Ribosome pathway were significantly downregulated in FAM99B-overexpressing and DDX21-silenced HCC cells (Fig. 4A, B). Since the ribosome is a component of the translation machinery, we hypothesized that FAM99B and DDX21 might regulate mRNA translation in HCC cells. To test this hypothesis, we performed surface sensing of translation (SUnSET), a method for monitoring overall protein synthesis by detecting puromycin-labeled polypeptides. The results revealed that global protein synthesis was increased after FAM99B knockout and that overexpression of FAM99B decreased global protein synthesis in HCC cells (Fig. 4C), but this decrease was reversed by overexpression of DDX21 (Fig. 4D). In addition, we performed an O-propargyl-puromycin (OP-Puro) assay to measure the incorporation of OP-Puro-tagged polypeptides. Fluorescence intensity in cells was analyzed using laser confocal microscopy or Fluorescence-Activated Cell Sorting (FACS). The results demonstrated that the overall translation activity in HepG2 cells with FAM99B knockout was significantly higher compared to the control cells. In contrast, overexpression of FAM99B resulted in a marked decrease in protein synthesis in Huh7 cells (Fig. 4E and Supplemental Fig. S5A), although restoration of DDX21 expression rescued this phenotype in Huh7 and HepG2 cells (Fig. 4F). Consistent with these findings, knockdown of DDX21 in Huh7 and HepG2 cells significantly reduced overall translation activity (Supplemental Fig. S5B–D). These results indicated that FAM99B suppressed protein translation in HCC cells by downregulating the expression of DDX21.

### FAM99B inhibits ribosome biogenesis by regulating rRNA processing and RPS29/RPL38 transcription via DDX21 in HCC cells

**Fig. 5:**
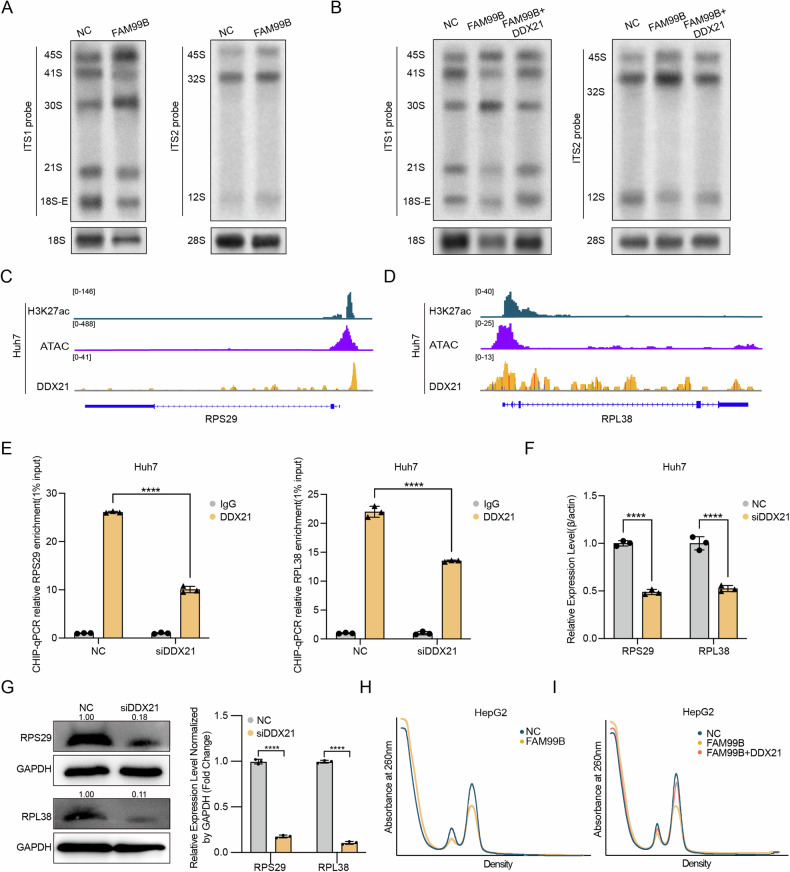
FAM99B inhibits ribosome biogenesis by regulating rRNA processing and RPS29/RPL38 transcription via DDX21 in HCC cells. **A** The expression levels of pre-rRNAs and mature rRNAs after FAM99B overexpression in Huh7 cells were measured via northern blotting. Intermediate forms of the mature 18 S rRNA (45 S, 30 S, 21 S, and 18 S-E pre-rRNA) and of the mature 28 S and 5.8 S rRNAs (45 S, 32 S and 12 S) were detected by the ITS1 and ITS2 probes, respectively. The mature 18S and 28S rRNAs were detected by the 18 S and 28S probes, respectively (*n* = 3 independent experiments). The rRNA processing pathway is described in Supplemental Fig. S7A. **B** The ITS1, ITS2, 18 S and 28 S probes were used to detect changes in pre-rRNA and mature rRNA levels after simultaneous overexpression of FAM99B and DDX21 in Huh7 cells via Northern blotting (*n* = 3 independent experiments). **C** Assay for transposase accessible chromatin followed by sequencing (ATAC-seq) and ChIP-seq data for H3K27ac and DDX21 showing peaks at the promoter regions of RPS29 in Huh7 cells. **D** ATAC-seq and ChIP-seq data for H3K27ac and DDX21 showing peaks at the promoter regions of RPL38 in Huh7 cells. **E** DDX21 occupancy at the promoters of RPS29 (left) and RPL38 (right) was quantified via ChIP‒qPCR in DDX21-knockdown Huh7 cells. Data are represented as the mean ± SD (*n* = 3). Two-way ANOVA with correction for multiple comparisons. **F** The mRNA levels of RPS29 and RPL38 in Huh7 cells after DDX21 knockdown were measured by qPCR. **G** The protein levels of RPS29 and RPL38 in Huh7 cells after DDX21 knockdown were measured by Western blotting, followed by statistical analysis. Data are represented as the mean ± SD (*n* = 3). One-way ANOVA with correction for multiple comparisons. **H** Ribosomal subunit levels of 40 S and 60 S were detected after FAM99B overexpression, based on ribosomal subunit profiles following puromycin-mediated dissociation (*n* = 3 independent experiments). **I** Ribosomal subunit levels of 40S and 60S were detected after the co-overexpression of FAM99B and DDX21, based on ribosomal subunit profiles following puromycin-mediated dissociation (*n* = 3 independent experiments). *p < 0.05, **p < 0.01, ***p < 0.001, ****p < 0.0001, and ns, not significant.

Next, we aimed to explore the mechanism by which FAM99B and DDX21 affect global protein synthesis in HCC cells. DDX21, which is localized in the nucleolus, can promote rRNA transcription, processing and modification [15]. To determine whether rRNA transcription and processing are affected by FAM99B and DDX21 in HCC cells, we examined the abundances of various pre-rRNAs and mature rRNAs by quantitative northern blotting in Huh7 cells. Compared with control cells, FAM99B-overexpressing cells exhibited accumulation of 47/45S pre-rRNA and 30S pre-rRNA but decreased signals of 21S and 18S-E pre-rRNA (Fig. 5A). These results indicated an additional defect in the initial cleavage of pre-rRNAs at the A site after overexpression of FAM99B (Fig. 5A and Supplemental Fig. S6A), whereas restoration of DDX21 expression rescued this phenotype (Fig. 5B). Similarly, the knockdown of DDX21 in Huh7 cells resulted in the accumulation of 47/45S pre-rRNA and 30S pre-rRNA but reductions in the 21S and 18S-E pre-rRNA signals (Supplemental Fig. S6B). These results indicate that FAM99B and DDX21 can regulate the processing of pre-rRNAs at the A site. Additionally, GSEA of the RNA-seq data from cells with FAM99B overexpression and DDX21 knockdown revealed that FAM99B and DDX21 regulate the expression of RPs at the transcriptional level. To determine whether FAM99B/DDX21 can regulate the levels of RPs, chromatin immunoprecipitation followed by sequencing (ChIP-seq) was performed, and the results revealed that DDX21 was abundant at the promoters of RPS29 and RPL38 (Fig. 5C–E). After DDX21 knockdown, DDX21 enrichment at the RPS29/RPL38 promoters was reduced, suggesting that DDX21 may regulate RPS29/RPL38 transcription (Supplemental Fig. S6C and Fig. 5E). Knockdown of DDX21 resulted in significant downregulation of the mRNA and protein expression of RPS29/RPL38 (Fig. 5F, G). These results indicate that DDX21 regulates the transcription of RPS29/RPL38.

Because FAM99B and DDX21 affect pre-rRNA processing and the expression of the RPs RPS29 and RPL38, we speculated that FAM99B and DDX21 can further influence ribosome biogenesis. By using puromycin to dissociate 80S and polysomal ribosomes into free 40S and 60S subunits [25], we performed polysome profiling and revealed that the 40S and 60S subunits in FAM99B-overexpressing cells and DDX21-silenced cells were decreased compared with those in the corresponding control cells (Fig. 5H and Supplemental Fig. S6C). DDX21 overexpression reversed the reductions in the 40S and 60S fractions caused by FAM99B overexpression (Fig. 5I). These results indicate that FAM99B affects ribosome biogenesis by regulating rRNA processing and RPS29/RPL38 transcription via DDX21 in HCC cells.

### The FAM99B^65-146^ fragment plays a suppressive role in HCC cells

**Fig. 6:**
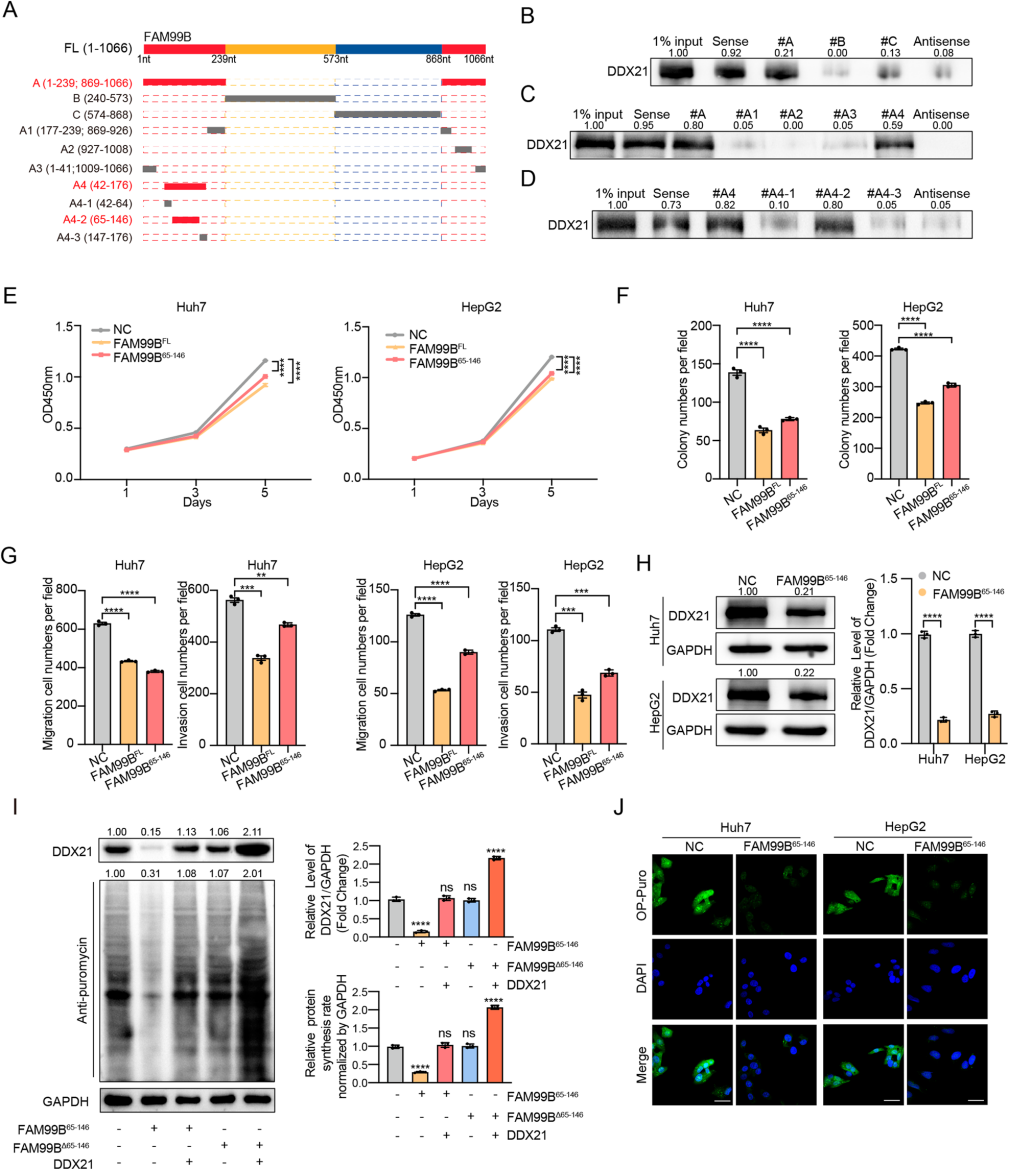
The FAM99B^65-146^ fragment plays a suppressive role in HCC cells. **A** Truncations of FAM99B were constructed on the basis of the secondary structure (Supplemental Fig. S8A). FAM99B was truncated to generate fragments (**A**) (1-239; 869-1066 nt), (**B**) (240-573 nt), and (**C**) (574-868 nt). Truncation A was further truncated to generate truncations A1 (177-239, 869-926 nt), A2 (927-1008 nt), A3 (1-41, 1009-1066 nt), and A4 (42-176 nt). Truncation A4 was further truncated to generate truncations A4-1 (42-64 nt), A4-2 (65-146 nt), and A4-3 (147-176 nt). **B**–**D** Western blot analysis of DDX21 expression in the samples precipitated with full-length FAM99B, FAM99B truncations ((**A**–**C**) **(B**), (A1, A2, A3, A4) (**C**), (A4-1, A4-2 and A4-4, A4) (**D**)) and the FAM99B antisense probe. **E** A CCK-8 assay was used to evaluate the proliferation ability of Huh7 and HepG2 cells following overexpression of FAM99B^65-146^. Data are represented as the mean ± SD (*n* = 3). Two-way ANOVA with correction for multiple comparisons. **F** Results of colony formation assays in HCC cells with FAM99B^65-146^ overexpression and a statistical histogram of colony formation data for three replicates. Data are represented as the mean ± SD (*n* = 3). Unpaired *t*-test. **G** Results of migration and invasion assays of Huh7 and HepG2 cells with FAM99B^65-146^ overexpression and statistical histogram of cell migration/invasion data for three replicates. Data are represented as the mean ± SD (*n* = 3). One-way ANOVA with correction for multiple comparisons. **H** The protein levels of DDX21 in Huh7 and HepG2 cells after overexpression FAM99B^65-146^ were measured by western blotting, followed by statistical analysis. Data are represented as the mean ± SD (*n* = 3). Unpaired *t*-test. **I** SUnSET was performed to evaluate the protein levels of DDX21 and protein synthesis rates under the following conditions in Huh7 cells: overexpression of FAM99B^65-146^, overexpression of FAM99B^Δ65-146^, co-expression of FAM99B^65-146^ and DDX21, and co-expression of FAM99B^Δ65-146^ and DDX21. The panel on the right represents the statistical analysis of the western blot. Data are represented as the mean ± SD (*n* = 3). One-way ANOVA with correction for multiple comparisons. **J** The effects of FAM99B^65-146^ overexpression on protein synthesis in Huh7 and HepG2 cells were evaluated via an OP-Puro assay. *p < 0.05, **p < 0.01, ***p < 0.001, ****p < 0.0001, and ns, not significant. Scale bar, 25 μm.

To explore the potential therapeutic role of FAM99B in HCC, we first aimed to identify the fragment of FAM99B that plays an inhibitory role in HCC cells. To identify the region of FAM99B that interacts with DDX21, we constructed 3 truncated fragments of FAM99B (fragment A: 1-239; 869-1066 nt, fragment B: 240-573 nt, fragment C: 574-868 nt) on the basis of the secondary structure predicted by RNAfold (http://rna.tbi.univie.ac.at/cgi-bin/RNAWebSuite/RNAfold.cgi) (Fig. 6A, Supplemental Fig. S7A, B). RNA pulldown experiments with the truncated FAM99B fragments A, B and C indicated that fragment A of FAM99B binds to DDX21 (Fig. 6B). Then, on the basis of the secondary structure of FAM99B, fragment A was further truncated to generate truncations A1, A2, A3 and A4 (fragment A1: 177-239; 869-926 nt, fragment A2: 927-1008 nt, fragment A3: 1-41; 1009-1066 nt, fragment A4: 42-176 nt). The results of RNA pulldown experiments with FAM99B truncations A1, A2, A3 and A4 indicated that the truncation A4 of FAM99B binds to DDX21 (Fig. 6C). Similarly, on the basis of the secondary structure, truncation A4 of FAM99B was further truncated to generate FAM99B truncations A4-1, A4-2, A4-3 and A4-4 (fragment #A4-1: 42-64 nt, fragment #A4-2: 65-146 nt, fragment #A4-3:147-176 nt). The results of RNA pulldown experiments indicated that truncation A4-2 of FAM99B binds to DDX21 (Fig. 6D). Thus, the fragment of FAM99B that interacts with DDX21 is represented by truncation A4-2 (hereafter referred to as FAM99B^65-146^). To confirm whether FAM99B^65-146^ inhibits the progression of HCC, we overexpressed FAM99B^65-146^ in Huh7 and HepG2 cells, and the results revealed that FAM99B^65-146^ inhibited the proliferation, colony formation, migration and invasion of Huh7 and HepG2 cells (Fig. 6E–G and Supplemental Fig. S7C, D). As expected, FAM99B^65-146^ was able to downregulate the protein levels of DDX21, thereby inhibiting ribosome biogenesis and protein synthesis in HCC cells (Fig. 6H–J, Supplemental Fig. S7E–G), whereas FAM99B^△65^^–146^ showed no such effects (Fig. 6I, Supplemental Fig. S7F, G). Thus FAM99B^65-146^ exhibiting activity similar to that of full-length FAM99B. These results indicate that FAM99B^65-146^ is the region of FAM99B that binds to DDX21 and that FAM99B^65-146^ can decrease the protein level of DDX21, inhibit protein synthesis, and play a suppressive role in HCC cells.

### GalNAc-FAM99B^65-146^ administration effectively attenuates the growth and metastasis of orthotopic xenograft tumors

**Fig. 7:**
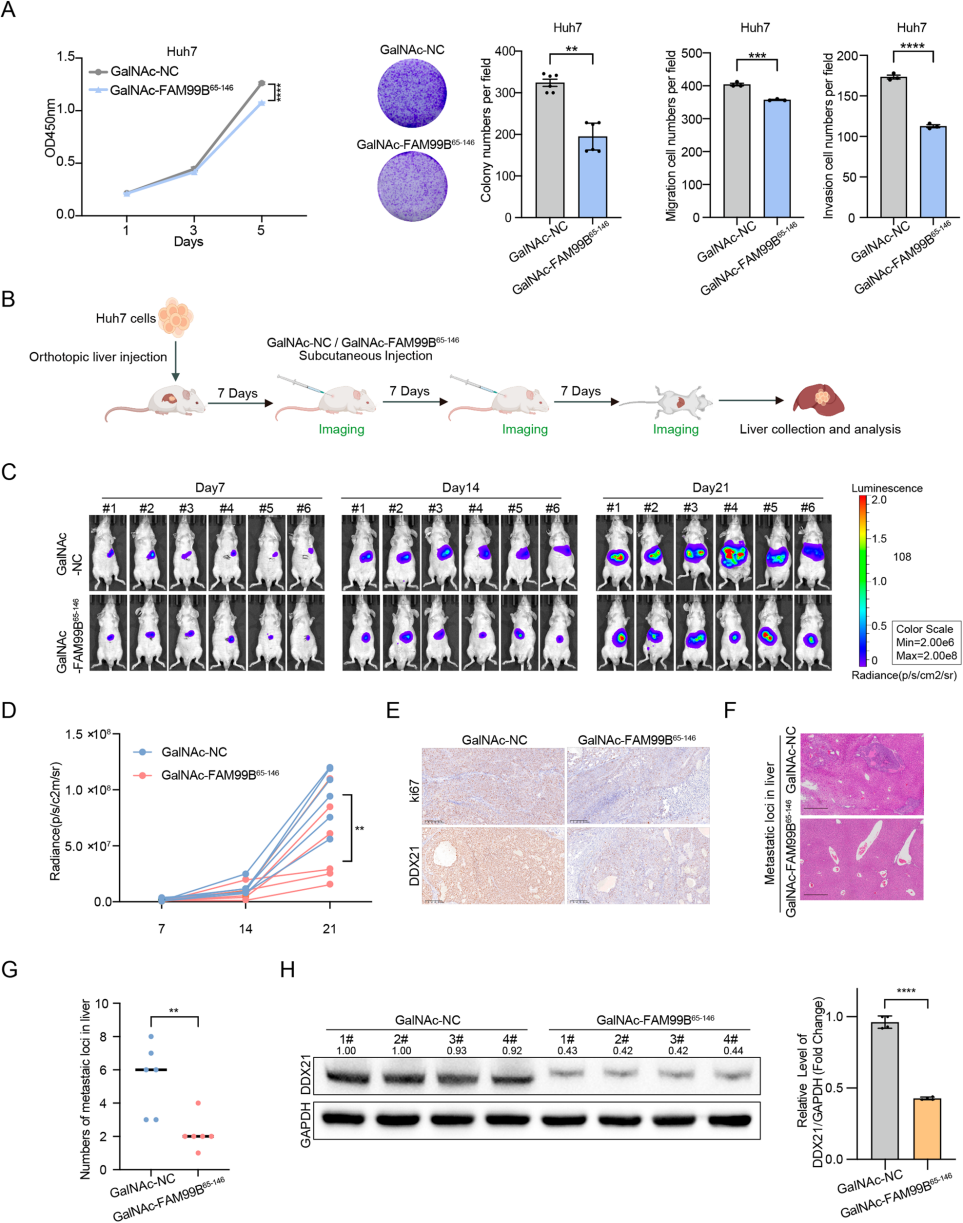
GalNAc-FAM99B^65-146^ administration significantly inhibits the growth and metastasis of orthotopic liver xenografts in nude mice. **A** GalNAc-NC and GalNAc-FAM99B^65-146^ were added to Huh7 cells for 48 h, then CCK-8, colony formation, migration, and invasion assays were performed, followed by statistical analysis. Data are represented as the mean ± SD (*n* = 3). One-way ANOVA or Two-way ANOVA with correction for multiple comparisons. **B** Establishment of the subcutaneous xenograft models and the frequency of treatment administration. Each BALB/c nude mouse was intrahepatically injected with 4 × 107 Huh7 cells, 7 days after orthotopic transplantation, the mice were randomly divided into two groups and were treated with GalNAc-NC or GalNAc-FAM99B^65-146^ (5 mg/kg) via subcutaneous injection weekly for 2 weeks, after which in vivo imaging was performed. **C** In vivo bioluminescence imaging of the nude mouse orthotopic liver xenograft model (*n* = 6). **D** Statistical analysis of the mean luminescence intensity in the in vivo images of the mice. Data are represented as the mean ± SD (*n* = 3). Unpaired *t*-test. **E** Representative images of Ki67 and DDX21 immunohistochemical staining in orthotopic liver xenograft tissues from mice in the GalNAc-NC and GalNAc-FAM99B^65-146^ groups. **F** Representative images of H&E staining in tissues from mice in the GalNAc-NC- and GalNAc-FAM99B^65-146^-treated groups. **G** Statistical analysis of metastatic foci in the GalNAc-NC and FAM99B^65-146^-treated groups. Data are represented as the mean ± SD (*n* = 6). Unpaired *t*-test. **H** Western blot analysis was performed to evaluate DDX21 expression in tumor samples from the GalNAc-NC and GalNAc-FAM99B^65-146^ treatment groups, followed by statistical analysis. Data are represented as the mean ± SD (*n* = 3). Unpaired *t*-test. *p < 0.05, **p < 0.01, ***p < 0.001, ****p < 0.0001, and ns, not significant. Scale bar: 200 μm.

GalNAc is a natural ligand of the asialoglycoprotein receptor (ASGPR), which is highly expressed on the membrane surface of hepatocytes and mediates clathrin-mediated endocytosis. Because GalNAc is an advanced platform for liver-targeted delivery, it is used in various RNA therapeutics, and several conjugates are in phase I-III clinical trials [16, 26, 27]. FAM99B is liver specific, and FAM99B^65-146^ inhibits the proliferation and metastasis of HCC cells in vitro. Therefore, the administration of GalNAc-FAM99B^65-146^ might constitute a novel strategy for the treatment of HCC. To test this hypothesis, we first conducted RT‒qPCR to examine the of GalNAc conjugation on the overexpression efficiency of FAM99B^65-146^ and found an overexpression efficiency of 25-fold (Supplemental Fig. S8A). Moreover, GalNAc-FAM99B^65-146^ inhibited the proliferation, colony formation, migration and invasion of Huh7 cells (Fig. 7A and Supplemental Fig. S8B). To investigate the therapeutic effects of GalNAc-FAM99B^65-146^ on HCC, we orthotopically injected Huh7 cells stably expressing Luciferase into the livers of BALB/c nude mice to establish an orthotopic xenograft model of HCC. The mice were subcutaneously administered two 5 mg/kg doses of GalNAc-FAM99B^65-146^ or GalNAc-NC (*n* = 6 mice/group) (Fig. 7B). We analyzed weekly imaging data from nude mice bearing orthotopic liver xenografts and found that the tumor area in the GalNAc-FAM99B^65-146^ treatment group was significantly smaller than that in the control group on day 21, indicating a lower tumor growth rate (Fig. 7C, D and Supplemental Fig. S8C). Consistent with this observation, staining for the cell proliferation marker Ki67 revealed that the expression of Ki67 was decreased in the GalNAc-FAM99B^65-146^ treatment group (Fig. 7E). Moreover, H&E staining revealed fewer intrahepatic metastases in the GalNAc-FAM99B^65-146^ treatment group (Fig. 7F, G). As expected, the protein level of DDX21 in the xenografts was significantly reduced after treatment with GalNAc-FAM99B^65-146^ (Fig. 7E and H). These results indicate that GalNAc-FAM99B^65-146^ treatment significantly inhibited the growth and metastasis of orthotopic tumor xenografts. Futhermore, we monitored the body weight of the mice from day 7 to day 21. The results showed that no significant weight loss in the treatment mice (Supplemental Fig. S8D). In addition, we analyzed the expression levels of FAM99B^65-146^ in the liver and lung tissues. The results revealed that FAM99B^65-146^ did not accumulate in the lung tissue, but was retained in the liver tissue (Supplemental Fig. S8E, F). These results can initially indicate that GalNAc-FAM99B^65-146^ does not cause observable toxicity in mice. In conclusion, these results indicate that GalNAc-FAM99B^65-146^ administration is a potential therapeutic strategy for HCC.

## Discussion

In this study, we identified lncRNAs specifically expressed in the liver and found that FAM99B is frequently downregulated and functions as a tumor suppressor in HCC. It has been reported that FAM99B inhibits HCC cell proliferation, migration, and invasion in vitro [11]. Another study revealed that FAM99B in exosomes derived from human umbilical cord mesenchymal stem cells (hucMSC-Exo) induced cell cycle arrest and apoptosis, inhibited HCC cell migration and invasion, and suppressed HCC tumorigenesis in vivo [28]. Our study revealed that FAM99B inhibited ribosome biogenesis and protein translation by regulating rRNA processing and RP transcription, thereby decreasing the proliferation, tumorigenicity, and metastasis of HCC cells in vitro and in vivo. Thus, our findings reveal a novel biological role of FAM99B in HCC.

FAM99B was predicted to be involved mainly in the “Metabolic pathways” and “Blood coagulation” pathways by analysis of coexpressed genes [11] and in the pathways related to the terms response to wounding and lipid biosynthetic process through enrichment analysis of FAM99B-binding proteins [12]. However, the molecular mechanisms of FAM99B in tumors have not been experimentally validated or further explored. In this study, we found that FAM99B can bind to DDX21 and decrease its protein expression level. FAM99B promotes the export of DDX21 from the nucleus by recruiting XPO1. Relatively large cargo molecules (>40 kDa) require active transport via transport receptors [29, 30]; these receptor proteins are classified as importins (for nuclear import), exportins (for nuclear export), and transportins (for both import and export) [31]. CRM1/Xpo1 is the most important and best characterized exportin [31, 32]. However, when XPO1 was knocked down, overexpression of FAM99B did not increase the translocation of DDX21 to the cytoplasm; instead, it remained in the nucleus. After export from the nucleus, DDX21 is cleaved at D126 via casp3/6 in the cytoplasm, leading to its degradation. It has been reported that DDX21 undergoes caspase-dependent cleavage after RNA virus infection or treatment with RNA/DNA ligands [24, 33], but the mechanism by which DDX21 is translocated to the cytoplasm has not been further explored. Here, we revealed that the protein level of DDX21 is decreased by the lncRNA FAM99B via casp3/6, suggesting the mechanism underlying the nuclear export of DDX21.

DDX21 is required for RNA helicase activity and controls multiple steps of ribosome biogenesis in human cells [17, 18, 34]. DDX21 is a component of the human UTP-B complex and is found in early and late preribosomal intermediates [35]. DDX21 interacts with SIRT7, which deacetylates DDX21 to increase its R-loop-unwinding activity and overcomes R-loop-mediated stalling of RNA polymerases [14]. Recently, it has been reported that DDX21 binds to the methyltransferase complex (MTC), facilitating the recruitment of the MTC to R-loops. The MTC, in turn, recruits the nuclease XRN2 to promote transcription termination. Failure to recruit the MTC via DDX21 leads to transcriptional readthrough, which results in DNA damage [36]. In the nucleolus, DDX21 occupies the transcribed rDNA locus, directly contacts both rRNAs and snoRNAs, and promotes rRNA transcription, processing and modification. In the nucleoplasm, DDX21 binds 7SK RNA and is recruited to the promoters of Pol II-transcribed genes encoding RPs and snoRNAs, thereby promoting the transcription of its target genes [15]. In addition, DDX21 forms ring-shaped structures surrounding multiple Pol I complexes and suppresses pre-rRNA transcription; moreover, the binding of the lncRNA SLERT allosterically alters individual DDX21 molecules, loosens the DDX21 ring, and evicts DDX21, relieving its suppression of Pol I transcription [37]. However, the biological function and mechanism of DDX21 in HCC have not been reported. Here, we found that DDX21 was regulated by the lncRNA FAM99B, which decreased the DDX21 protein level. DDX21 inhibited rRNA processing and RPS29/RPL38 transcription, resulting in decreased assembly of the 40 S and 60 S ribosomal subunits and the inhibition of ribosome biogenesis, thereby further reducing overall protein synthesis in HCC cells.

The natural receptor for GalNAc is highly expressed in hepatocytes. GalNAc is a clinically advanced platform, convenient to administer, and stably metabolized; thus, it has broad applicability in RNAi delivery for cancer therapy [38, 39]. The GalNAc–siRNA conjugates givosiran, which is used to treat acute intermittent hepatic porphyria [40], and lumasiran [41], which is used to treat primary hyperoxaluria type 1, have been approved by the FDA. Inclisiran is used to treat hypercholesterolemia or mixed dyslipidemia by inhibiting the hepatic synthesis of proprotein convertase subtilisin–kexin type 9 (PCSK9) [42, 43]. In the present study, the vast majority of studies using lncRNA for cancer regard it as a target for cancer therapy. GalNAc-conjugated siRNA-mediated inhibition of lncRNA expression was used to treat cancer. For example, there was a study showed that GalNAc-conjugated siRNAs targeting lncRNA16 can restore chemosensitivity and inhibit tumor growth, and combining first-line platinum-based chemotherapy with lncRNA16 interference can significantly increase the antitumor efficacy [44]. GalNAc–siURB1-AS1 specifically inhibited the expression of the lncRNA URB1-AS1 in an in vivo tumor model and significantly sensitized sorafenib-resistant HCC cells to sorafenib and inhibited tumor growth [45]. It is worth noting that a study on the treatment of phenylketonuria (PKU) used lncRNA as a therapeutic agent, which reported that GalNAc-labeled lncRNA HULC mimics treatment reduced the excess phenylalanine (Phe) concentration in mice, providing a potential intervention measure for PKU patients [46]. In our study, GalNAc-FAM99B^65-146^ inhibited the growth and metastasis of orthotopic HCC xenografts. These results indicate that GalNAc-FAM99B^65-146^ has potential therapeutic utility in HCC. Our study provides a new approach for the application of lncRNAs in gene therapy and reveals a potential strategy for the treatment of HCC. This is the first report of the use of a lncRNA as an agent rather than a target in tumor treatment.

**Fig. 8:**
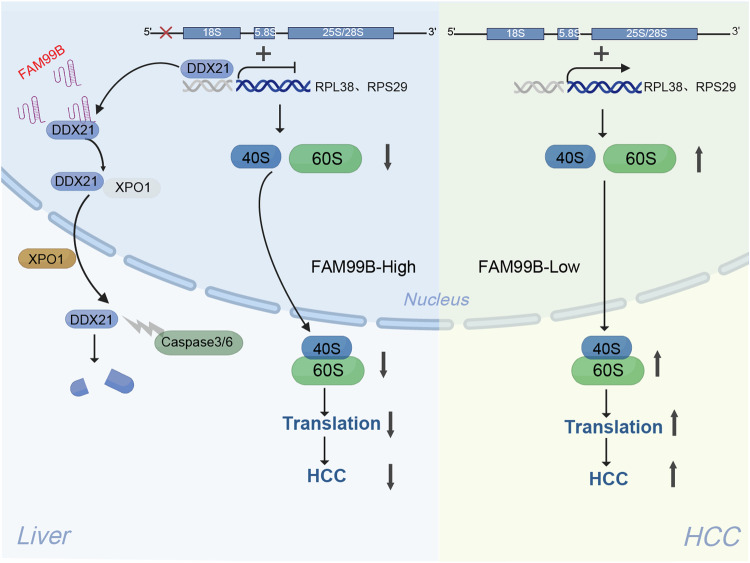
Graphical illustration of the mechanism of FAM99B in HCC.

In conclusion, FAM99B is a liver-specific lncRNA and is downregulated in HCC. FAM99B binds to DDX21 and promotes the nuclear export of DDX21 by interacting with XPO1. DDX21 is degraded via caspase3/6-mediated cleavage at D126 in the cytoplasm. FAM99B inhibits rRNA processing and RPS29/RPL38 transcription via DDX21, leading to reduced ribosome biogenesis and decreased protein synthesis, resulting in inhibition of the proliferation and metastasis of HCC cells in vivo and in vitro. Notably, the administration of GalNAc-FAM99B^65-146^ effectively inhibited the growth and metastasis of orthotopic xenograft tumors in vivo, revealing a promising strategy for the treatment of HCC (Fig. 8).

## Methods

### In vivo assays

Female athymic BALB/c nude mice, aged 6 weeks, were injected subcutaneously with 0.2 ml of a suspension containing 1 × 10^6^ Huh7 cells (pWPXL-VECTOR or pWPXL-FAM99B) in the right axilla. The tumor growth rate was monitored, and the tumor volume was calculated according to the following formula: volume = length × width^2^ × 0.5. To further investigate the effect of FAM99B on tumor invasion in vivo, we established a metastasis model in nude mice by injecting 1 × 10^6^ Huh7 cells (pWPXL-VECTOR or pWPXL-FAM99B) into the liver. Seven weeks later, the mice were sacrificed, and the livers were harvested.

### RNA-seq and GSEA

RNA from cells with stable FAM99B overexpression and DDX21 knockdown was extracted with TRIzol reagent (Invitrogen, CA, USA), the RiboMinus Eukaryote Kit (QIAGEN, CA, USA) was used to remove rRNA, and the NEBNext Ultra Directional RNA Library Prep Kit (New England Biolabs, MA, USA) was used to construct the RNA-seq library. The read count of each gene was normalized Fragments Per Kilobase of transcript per Million mapped reads (FPKM) method. The fold change in the expression level in the control group compared with the overexpression/knockdown group was calculated for each target gene, and genes with a fold change in expression >1.5 after FAM99B overexpression or <0.67 after DDX21 knockdown were imported for GSEA.

### SUnSET method

HCC cells were treated with 10 μg/ml puromycin (New England Biolabs, MA, USA; Biotech, Shanghai, China) at 37 °C in an incubator with 5% CO_2_ for 30 min and were then lysed in SDS loading buffer (Epizyme, Shanghai, China). Cell lysates were analyzed via western blotting with an anti-puromycin antibody.

### Polysome profiling

HCC cells were treated with 100 μg/ml cycloheximide (CHX; Sangon Biotech, Shanghai, China) at 37 °C with 5% CO_2_ for 3 min. Lysis buffer was added to the cells for 30 min, and the cells were then incubated with RNase I (Vazyme, Nanjing, China) and DNase I (NEB, UK) for 45 min. The monomers were separated by sucrose cushion centrifugation at 50,000 rpm for 2 h at 4 °C. The absorbance of each sample was measured at 260 nm.

### OP-Puro assay

The OPP assay was performed with a Click-iT Plus OPP Alexa Fluor 488 protein synthesis test kit (Thermo Fisher Scientific, Carlsbad, California, USA) according to the manufacturer’s instructions.

### In vivo assays

For the subcutaneous tumor formation assay in nude mice, Huh7 cells (1 × 10^6^) with NC or FAM99B overexpression were injected subcutaneously into 6-week-old female BALB/c nude mice. Four weeks later, the mice were sacrificed, and the livers were harvested.

For the orthotopic xenograft assay, Huh7 cells (1 × 10^6^) with NC or FAM99B overexpression were injected into the livers of 6-week-old female BALB/c nude mice. One month later, the mice were sacrificed, and the livers were harvested.

For GalNAc-RNA treatment in the liver orthotopic xenograft model: GalNAc-RNA was synthesized by Hippobio (Huzhou, China). The sequence of NC in GalNAc-NC is: GCCUGCCCCUCGCGCAUCCGGGUGCUACUGGAGGAGCGGGAGCGGGAAAUAGGUGCCGCAGGCCCAGCCGCACAGAAUGGCA. The sequence of FAM99B^65-146^ in GalNAc-FAM99B^65-146^ is GGCUGUGUGGCCCCAGCUCCCUG AGCCCAGAGGGAGGUGAGGGUGAGAAGGCCUGGACCAGGCAGGACGCAGCCCCCAGGGC. The GalNAc-RNA conjugate was modified with dioxymethyl and a fluoro group to increase its stability in vivo, and the 3’ end was modified with GalNAc. Each BALB/c nude mouse was intrahepatically injected with 4 × 10^7^ Huh7 cells (with stable expression of EGFP-Luciferase) to establish orthotopic xenografts. Seven days after orthotopic transplantation, the mice were randomly divided into two groups according to the bioluminescence intensity and were treated with GalNAc-NC or GalNAc-FAM99B^65-146^ (5 mg/kg) via subcutaneous injection weekly for 2 weeks, after which in vivo imaging was performed. At the end of the third week, the mice were euthanized, and their livers were harvested and photographed. The tumor tissues were retained for RNA and protein extraction, and the mouse liver and lung tissues were fixed with 4% paraformaldehyde for subsequent H&E staining and immunohistochemical (IHC) staining.

### Statistical analysis

All the statistical analyses were performed via GraphPad Prism software (version 9.0). Student’s *t*-test was used to determine the significance of differences between two groups; one-way ANOVA was used to determine the significance of differences based on one variable among multiple groups; two-way ANOVA was used to determine the significance of differences based on two variables among multiple groups. A value of *p* < 0.05 was considered to indicate statistical significance, which was denoted as follows: *p < 0.05, **p < 0.01, ***p < 0.001, ****p < 0.0001. The abbreviation “ns” indicates statistical insignificance.

## Supplementary information

supplementary information

Table S1

Table S2

Full and uncropped western blots

## Data Availability

Data supporting the findings of the current study are available in the NCBI Gene Expression Omnibus (accession no: GES271217).
