# Correction: The liver-specific long noncoding RNA FAM99B inhibits ribosome biogenesis and cancer progression through cleavage of dead-box Helicase 21

**DOI:** 10.1038/s41419-025-07681-2

**Published:** 2025-05-06

**Authors:** Yifei He, Hongquan Li, Qili Shi, Yanfang Liu, Qiaochu Pan, Xianghuo He

**Affiliations:** 1 Fudan University Shanghai Cancer Center and Institutes of Biomedical Sciences; Department of Oncology, Shanghai Medical College, Fudan University, Shanghai, 200032 China; 2 Key Laboratory of Breast Cancer in Shanghai, Fudan University Shanghai Cancer Center, Fudan University, Shanghai, 200032 China

**Keywords:** Targeted therapies, Mechanisms of disease

Correction to: *Cell Death & Disease* 10.1038/s41419-025-07401-w, published online 14 February 2025

In this article, the image for the FAM99B group in Supplementary Figure S2E, which depicts the invasion assay of Huh7 cells overexpressing FAM99B, has been revised.
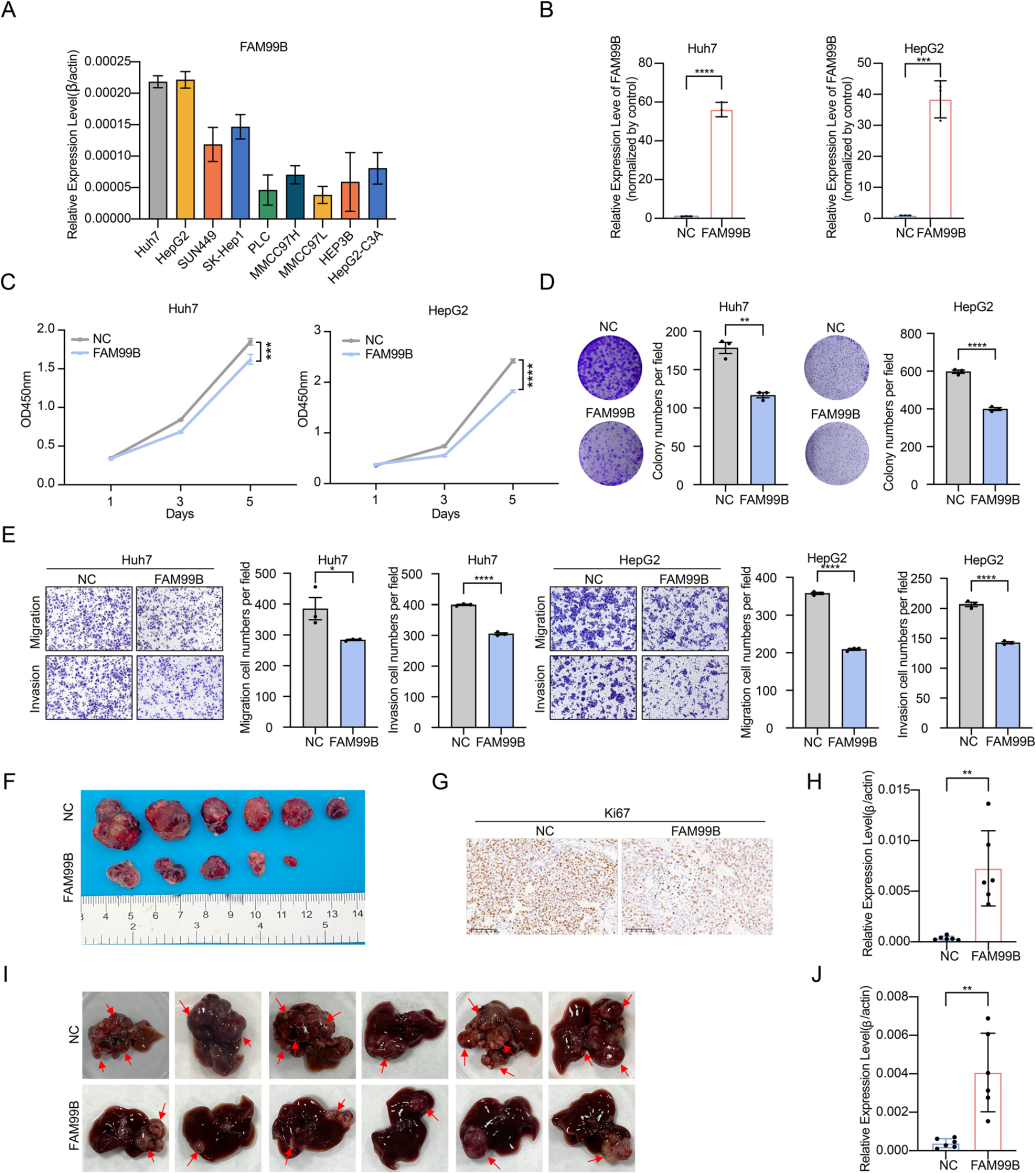


The original article has been corrected.

## Supplementary information

Supplementary Information

