# Supplementary figures and images for: The liver-specific long noncoding RNA FAM99B inhibits ribosome biogenesis and cancer progression through cleavage of dead-box Helicase 21
Source: Cell Death Dis. 2025 Feb 14;16(1):97. doi: 10.1038/s41419-025-07401-w (PMC11829061; doi:10.1038/s41419-025-07401-w)

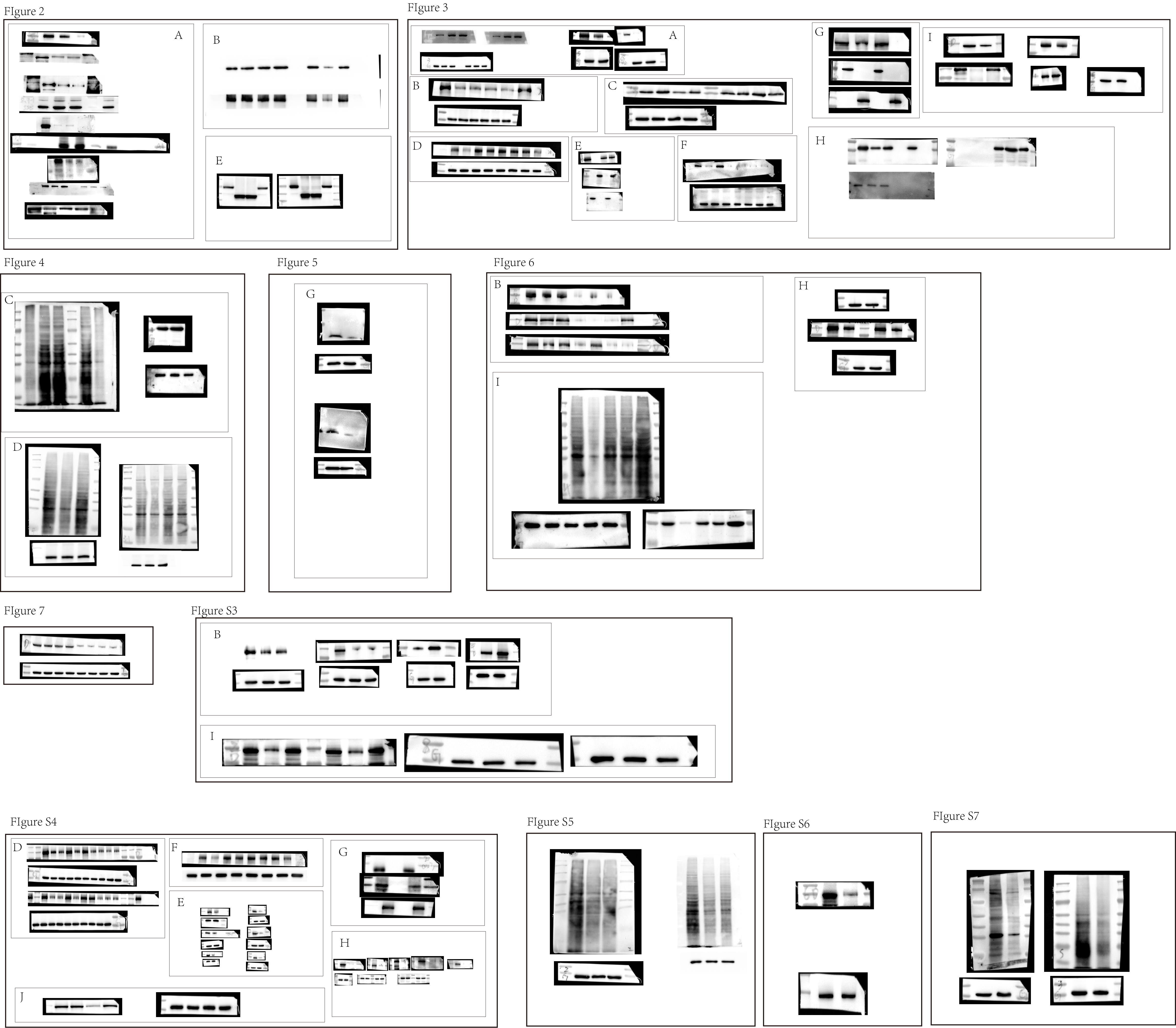

Supplement: Supplementary file 4 — Full and uncropped western blots [file 41419_2025_7401_MOESM4_ESM.png]
