# Supplementary material for: Correction: The liver-specific long noncoding RNA FAM99B inhibits ribosome biogenesis and cancer progression through cleavage of dead-box Helicase 21
Source: Cell Death Dis. 2025 May 6;16(1):363. doi: 10.1038/s41419-025-07681-2 (PMC12056207; doi:10.1038/s41419-025-07681-2)
Supplement: Supplementary file 1 — Supplementary Information [file 41419_2025_7681_MOESM1_ESM.pdf]

Supplementary Information

The Liver-Specific Long Noncoding RNA FAM99B Inhibits Ribosome Biogenesis and Cancer Progression through Cleavage of Dead-Box Helicase 21

Yifei He[1], Hongquan Li[1,*], Qili Shi[1], Yanfang Liu[1], Qiaochu Pan[1], Xianghuo He[1,2,*]

[1]Fudan University Shanghai Cancer Center and Institutes of Biomedical Sciences; Department of Oncology, Shanghai Medical College, Fudan University, Shanghai 200032, China

[2]Key Laboratory of Breast Cancer in Shanghai, Fudan University Shanghai Cancer Center, Fudan University, Shanghai 200032, China

These authors contributed equally to this work: Yifei He and Hongquan Li.

[*]Corresponding Authors:

Xianghuo He, Email: xhhe@fudan.edu.cn; Hongquan Li, E-mail: hongquan@fudan.edu.cn, Fudan University Shanghai Cancer Center and Institutes of Biomedical Sciences; Shanghai Medical College, Fudan University, Rm. 302, Bldg. #7, 270 Dong An Road, Shanghai 200032, China. Tel: 86-21-34777329; Fax: 86-21-64172585.

Supplementary Materials and Methods

5' and 3' rapid amplification of cDNA ends (RACE)

The transcriptional initiation and termination of FAM99B were evaluated by 5' RACE and 3' RACE, respectively, with a SMARTer@RACE 5'/3' Kit (TaKaRa, Tokyo, Japan) in accordance with the user manual. The sequences of the gene-specific PCR primers used for 5' and 3' RACE are listed in Supplemental Table S2. The amplified products were gel purified and were then inserted into the pGEM-T vector. Multiple isolated colonies were picked and confirmed by sequencing.

Subcellular fractionation

HCC cells were lysed with buffer and incubated on ice for 10 min. After centrifugation, the supernatant was used for isolation of the cytoplasmic fraction, and the nuclear fraction was isolated via precipitation. RNA expression was analyzed, with β-actin used as the endogenous control for cytoplasmic RNA and the U2 small nuclear RNA as the endogenous control for nuclear RNA. Protein levels were determined with GAPDH as the cytoplasmic endogenous control and LAMIN B1 as the nuclear endogenous control.

RNA isolation, quantitative PCR (qPCR), and reverse transcription PCR (RT–PCR)

Total RNA from HCC samples or cell lines was isolated with TRIzol Reagent (Thermo Fisher Scientific, Carlsbad, California, USA), and RT–PCR was then performed with the PrimeScript RT Reagent Kit (TaKaRa, Tokyo, Japan). qPCR was performed with SYBR Premix (TaKaRa, Tokyo, Japan) to determine relative RNA expression levels. β-Actin was used as an internal control to quantify the mRNA levels of FAM99B and other genes. Relative RNA expression levels were calculated via the comparative Ct method. The primers used are listed in Supplemental Table S2.

Cell lines and reagents

HepG2, SK-Hep1, HepG2-C3A and HEK293T cells were obtained from the American Type Culture Collection (ATCC; Manassas, Virginia, USA). Huh7, MHCC-97L, MHCC-97H, HCCLM3 and SNU-449 cells were purchased from the Shanghai Cell Bank Type Culture Collection Committee (CBTCCC; Shanghai, China). All the cell lines were cultured in DMEM supplemented with 10% fetal bovine serum at 37 °C in 5% CO_2.

CRISPR/Cas9-mediated knockout (ko)

FAM99B knockout cell lines were generated via CRISPR/Cas9 genome editing system. The single guide RNAs (sgRNAs) were designed on the basis of the FAM99B sequence with CRISPR-ERA (http://crispr-era.stanford.edu/), and high-scoring sgRNAs were selected. The sequences are listed in Supplemental Table S2. The three candidate

sequences (sgRNA#1-#3) were subsequently cloned and inserted into the lenti-gRNA-puro vector. Combinations of paired sgRNAs were used to achieve the best efficiency. The “#1 + #2” sgRNA pair (sgFAM99B-Mix-1) and the “#2 + #3” sgRNA pair (sgFAM99B-Mix-2) were shown to be effective in both knockout systems.

RNA interference and production of lentiviral particles

The sequences of the small interfering RNA (siRNA) oligonucleotides targeting DDX21, CASP2, CASP3, CASP4, CASP6, CASP7, CASP8, CASP9, CASP10 and XPO1 are listed in Supplemental Table S2; the siRNA constructs were purchased from RiboBio (RiboBio Biotechnology, Guangzhou, China). The FAM99B and FAM99B fragments were subsequently cloned and inserted into the BamHI and EcoRI sites in the lentiviral expression vector pWPXL. The DDX21 expression vectors were constructed by inserting the corresponding open reading frame sequences into the pCDH-CMV lentiviral vector, and the DDX21 expression vectors were constructed in the pCMV-Flag lentiviral vector. The primers used for PCR are listed in Supplemental Table S2. For lentivirus production, the lentiviral vector, packaging plasmid (pAX2), and VSV-G envelope plasmid (pMD2.G) were cotransfected into HEK-293T cells with Lipofectamine 2000 (Thermo Fisher Scientific, Carlsbad, California, USA). The supernatants were collected 48 h after transfection and used to infect the corresponding HCC cells. The pAX2 and pMD2.G plasmids were gifts from Dr. Didier Trono.

Cell proliferation assay and colony formation assay

Cell viability was assessed via a Cell Counting Kit-8 (CCK-8) (MCE) assay. In a 96-well plate, 1500 cells were seeded per well, with 3 replicate wells per sample. The next day, the medium was replaced with complete medium containing 10% CCK-8 reagent, and the cells were incubated in a cell culture incubator for 1.5 h. The absorbance was then measured at 450 nm. For the colony formation assay, 2000 cells per well were seeded in a 6-well plate. After 10–14 days, the cells were stained with 0.1 mg/ml crystal violet at room temperature for 10 minutes and imaged with a scanner.

Migration and invasion assays

For the invasion assay, Millicell chambers were placed in a 24-well plate, and the membrane was coated with 30 μg of Matrigel (BD, USA). A total of 5×10^4 Huh7 cells or 8×10^4 HepG2 cells in serum-free medium were added to each Millicell chamber. We added DMEM containing 10% fetal bovine serum (FBS) to each lower chamber as a chemoattractant. After 16 h (Huh7) or 48 h (HepG2) in the incubator, the cells that migrated through the membranes were fixed with methanol and stained with crystal violet. We observed the cells under an inverted microscope and photographed them. The migration assay was conducted similarly but without a Matrigel coating on the membranes.

RNA immunoprecipitation (RIP) assay

HCC cells were collected, and 1 ml of lysis buffer (Thermo Fisher Scientific, Carlsbad, California, USA), 20 μl of protease inhibitor (Bimake, Houston, Texas, USA), and 10 μl of RNase inhibitor (Vazyme, Nanjing, China) were added to precipitate the cells. The mixture was incubated with rotation at 4°C for 2 h and was then centrifuged at 14,000 × g for 15 minutes. An anti-DDX21 antibody was added to the lysate, which was subsequently incubated on a rotator at 4°C overnight. Then, 30 μl of Protein G Dynabeads (Thermo Fisher Scientific, Carlsbad, California, USA) was added to the protein–antibody mixture, which was incubated with rotation at 4°C for 2 h. The beads were washed 6 times with NT2 buffer, and the RNA bound to the beads was then extracted with TRIzol, diluted in an equal volume of nuclease-free water and reverse transcribed into cDNA with PrimeScript RT Master Mix (TaKaRa, Tokyo, Japan). qPCR was conducted to evaluate the enrichment of FAM99B.

IP assay

HCC cells were lysed with IP lysis buffer and centrifuged to harvest protein. The appropriate antibody was incubated with protein G magnetic beads for 30 min at room temperature. The antibody-conjugated magnetic beads were then added to the lysate, which was subsequently incubated overnight at 4 °C. The unbound antibodies were subsequently removed by washing with NT2 buffer (50 mM Tris-HCl (pH 7.4), 150 mM NaCl, 1 mM $MgCl_2$, 0.05% NP-40). After washing, the proteins bound to the

beads were separated via sodium dodecyl sulfate–polyacrylamide gel electrophoresis (SDS–PAGE).

Western blot analysis

Western blot analysis was performed according to a standard protocol. in brief, proteins extracted from organoids or cell lines were separated via 10–12.5% SDS–PAGE and electrotransferred onto 0.2-μm nitrocellulose membranes (GE, CT, USA). After blocking for 1 h in 2.5% skim milk, the membranes were incubated with primary antibodies at 4°C overnight and were then incubated with secondary antibodies. Immune complexes were detected with a hypersensitive ECL kit (Biotend, Shanghai, China). Information on the antibodies used is provided in Supplemental Table S2.

RNA pulldown assay

To identify protein binding pairs, RNA pulldown was performed. Sense-FAM99B and antisense-FAM99B RNA probes were synthesized by transcription with T7 RNA polymerase (New England Biolabs, MA, USA) followed by labeling with Biotin RNA Labeling Mix (Roche, Indianapolis, Indiana, USA). The pretreated biotinylated RNA and 1 ml of protein lysate from HCC cells were incubated overnight on a rotator at 4°C. Then, 30 μl of streptavidin beads (Invitrogen, CA, USA) was added to 1 ml of the RNA–protein mixture, which was subsequently incubated on a rotator at 4 °C for 3 h. The beads were then washed 5 times in NT2 buffer. Proteins were precipitated, diluted

in 50 μl of protein lysis buffer, and analyzed via MS (Omicsolution, Shanghai, China). The primers used for the pulldown assay are listed in Supplemental Table S2.

RNAscope fluorescence in situ hybridization (FISH)

A FISH probe was synthesized and a fluorescence *in situ* hybridization kit (RiboBio Biotechnology, Guangzhou, China) was used for this assay. Cells were seeded at a density of 50%–70%, fixed with 4% formaldehyde at room temperature for 10 min, and permeabilized with 0.3% Triton X-100 for 20 min. The cells were blocked at room temperature for 30 minutes, prehybridized at 37°C for 30 minutes, and incubated with FISH Probe Mix overnight at 37°C in the dark. Posthybridization washes were performed to reduce the signal background, DAPI staining was performed, and the slides were mounted. Observation and imaging were conducted with a confocal laser scanning microscope.

ChIP

Cultured cells were subjected to crosslinking with 1% formaldehyde, and crosslinking was stopped by adding glycine to a final concentration of 0.125 M. The cells were collected and resuspended in ChIP lysis buffer containing protease inhibitors (Bimake, Houston, Texas, USA). Chromatin was sheared into 200–500 bp fragments via sonication under the appropriate conditions. ChIP-grade antibodies were added to protein A/G magnetic beads (Bimake, Houston, Texas, USA), and the mixture was incubated with rotation at room temperature for 30 minutes. Then, the chromatin

mixture was added to the beads, and the samples were incubated with rotation overnight at 4°C. The nonspecific fragments were removed, and the DNA was recovered. The primers used in the ChIP assay are listed in Supplemental Table S2.

Northern blot analysis

We used an Ambion NorthernMax Kit (Thermo Fisher Scientific, Carlsbad, California, USA) and a DIG Northern Starter Kit (Roche, Indianapolis, Indiana, USA) with digoxin-labeled RNA probes to detect FAM99B and pre-rRNAs. The primer and probe sequences are listed in Supplemental Table S2.

Immunofluorescence

Cells were treated as described for FISH and were then fixed, permeabilized and blocked. Then, the cells were incubated with a primary antibody overnight at 4°C. The next day, the slides were washed with PBS and incubated with a fluorescent secondary antibody in the dark for 1 h at room temperature prior to DAPI staining and mounting. Observation and imaging were conducted with a confocal laser scanning microscope. Information on the antibodies used is provided in Supplemental Table S2.

Supplemental Figures and Figure Legends

Supplementary Figure S1.

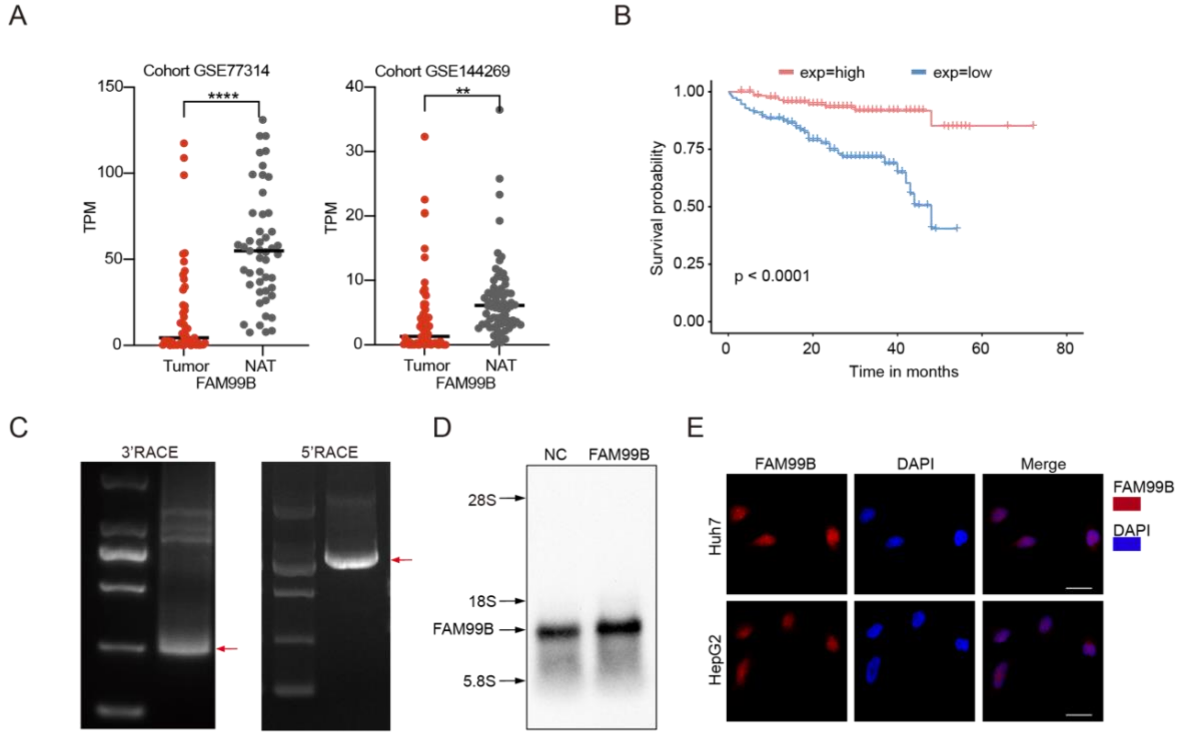

Figure S1. Characteristic features of FAM99B.

A. Relative expression of FAM99B in HCC tissues and normal tumor-adjacent tissues in the GSE77314 dataset (left) and GSE144269 dataset (right). Data are represented as the mean ± SD (n = 3). Unpaired t test. **B.** Kaplan–Meier analysis of overall survival in HCC patients in the ICGC cohort. **C.** Genomic location of FAM99B. **D.** Evaluation of full-length FAM99B by RACE. Representative images of the PCR products generated via 5‘RACE and 3’RACE. **E.** Northern blot analysis was performed to detect FAM99B in Huh7 cells. **F.** Subcellular localization of FAM99B, as determined by immunofluorescence staining, in Huh7 and HepG2 cells. *p < 0.05, **p < 0.01, ***p < 0.001, ****p < 0.0001. Scale bar, 25 µm.

Supplementary Figure S2

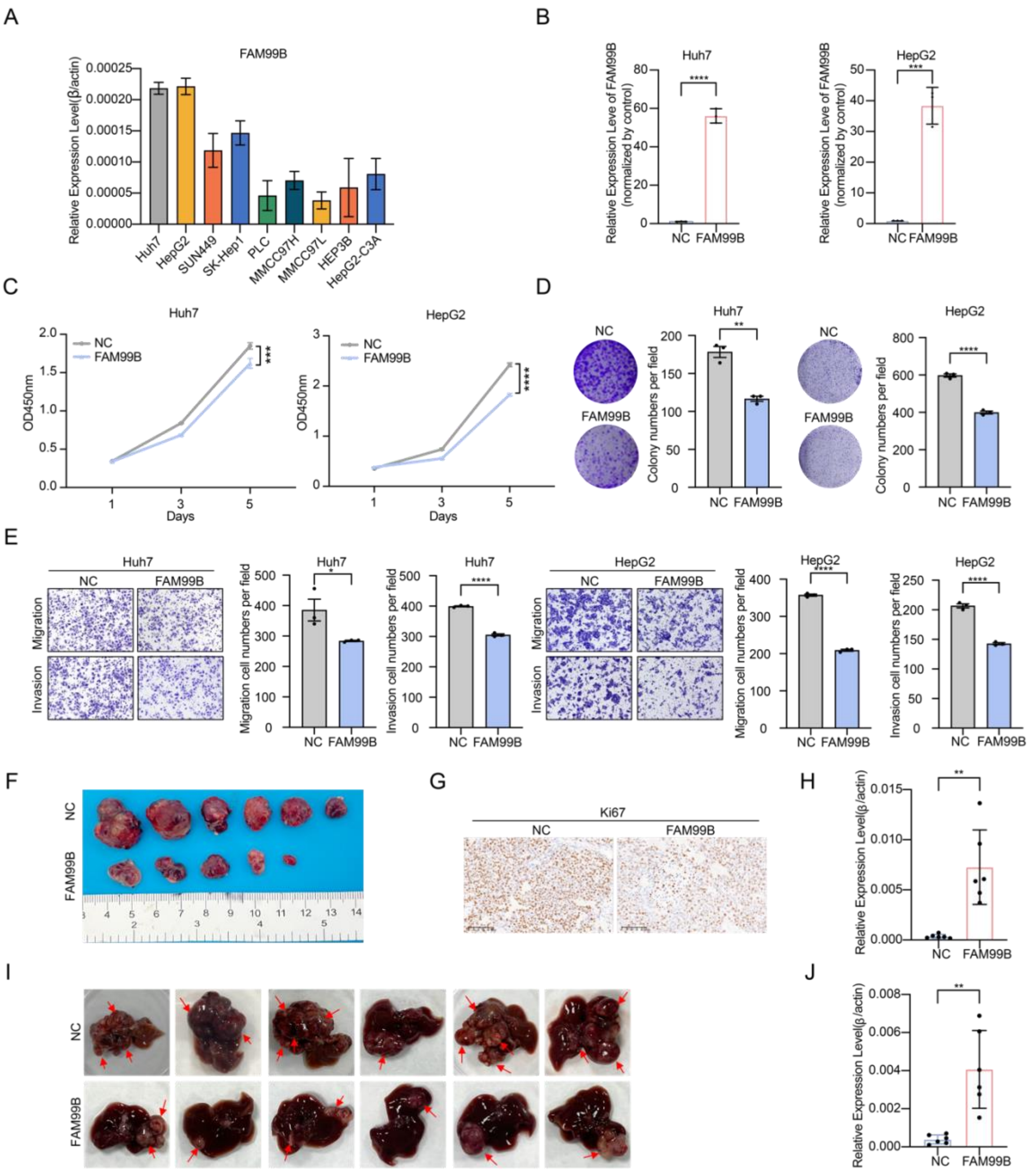

Fig S2. FAM99B inhibits the proliferation and metastasis of HCC cells *in vitro* and *in vivo*.

A. The expression level of FAM99B in different hepatoma cell lines was measured by qPCR. Data are represented as the mean ± SD (n = 3). **B.** The overexpression efficiency

of FAM99B in HCC cells was determined by qPCR. The mean ± s.d. Data are represented as the mean ± SD (n = 3). Unpaired t test. **C.** CCK-8 assay was used to evaluate the proliferation ability of HCC cells after FAM99B overexpression. Data are represented as the mean ± SD (n = 3). Two-way ANOVA with correction for multiple comparisons. **D.** Results of colony formation assays of HCC cells with FAM99B overexpression (left) and a statistical histogram of the colony formation data from three replicates (right). Data are represented as the mean ± SD (n = 3). Unpaired t test. **E** Migration and invasion assays of HCC cells with FAM99B overexpression (left) and a statistical histogram of the migration data from three replicates (right). Data are represented as the mean ± SD (n = 3). Unpaired t test. **F.** Growth of subcutaneous xenografts formed from pWPXL-FAM99B cells or vector cells in nude mice. **G.** Representative IHC images indicating Ki67 expression in mouse subcutaneous tumors. **H.** FAM99B expression in subcutaneous tumors from mice in the control group and mice in the FAM99B overexpression group. Data are represented as the mean ± SD (n = 3). Unpaired t test. **I.** Growth of orthotopic liver tumors formed from pWPXL-FAM99B cells or vector cells. **J.** FAM99B expression in orthotopic liver tumors from mice in the control group and the FAM99B overexpression group. Data are represented as the mean ± SD (n = 3). Unpaired t test. *$p < 0.05$, **$p < 0.01$, ***$p < 0.001$, ****$p < 0.0001$. Scale bar, 500 μm.

Supplementary Figure S3.

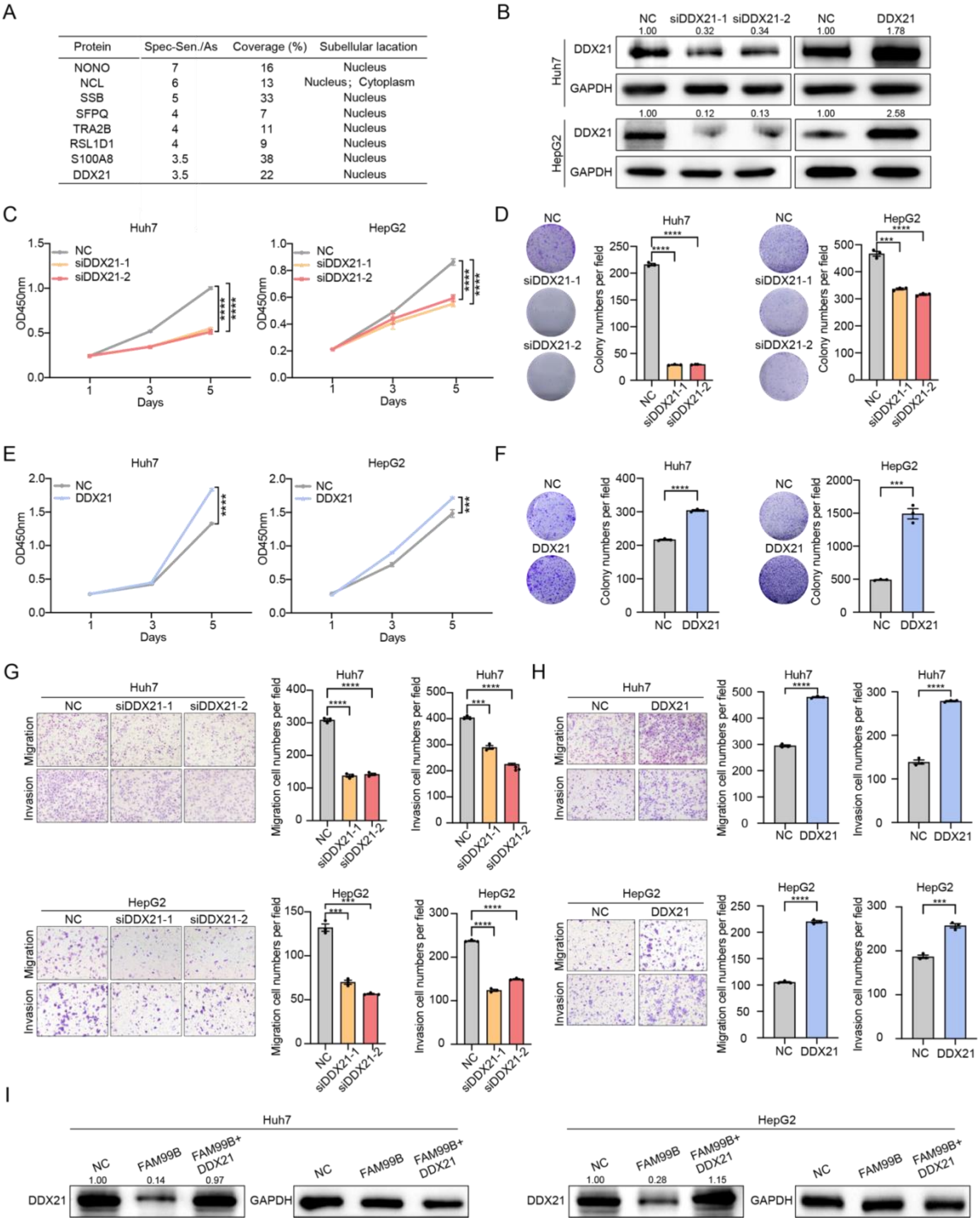

Figure S3. DDX21 inhibited the proliferation, migration and invasion of hepatocellular carcinoma cells.

A. Filtered list of precipitated proteins identified by mass spectrometry. **B.** The overexpression efficiency and knockdown efficiency of DDX21 in Huh7 and HepG2 cells were determined via Western blotting. (**C, E)** A CCK-8 assay was used to evaluate the proliferation ability of Huh7 and HepG2 cells following DDX21 knockdown (**C)** or overexpression (**E**). Data are represented as the mean ± SD (n = 3). Two-way ANOVA with correction for multiple comparisons. (**D, F)** Colony formation assays of Huh7 and HepG2 cells following DDX21 knockdown (**D)** or overexpression (**F**). Data are represented as the mean ± SD (n = 3). Unpaired t test. **G.** Migration and invasion assays of Huh7 and HepG2 cells following DDX21 knockdown. Data are represented as the mean ± SD (n = 3). One-way ANOVA with correction for multiple comparisons. **H.** Migration and invasion assays of Huh7 and HepG2 cells following DDX21 overexpression. Data are represented as the mean ± SD (n = 3). Two-way ANOVA with correction for multiple comparisons. **I.** After simultaneous overexpression of FAM99B and DDX21, the overexpression efficiency of DDX21 was determined by Western blotting. *$p < 0.05$, **$p < 0.01$, ***$p < 0.001$, ****$p < 0.0001$.

Supplementary Figure S4.

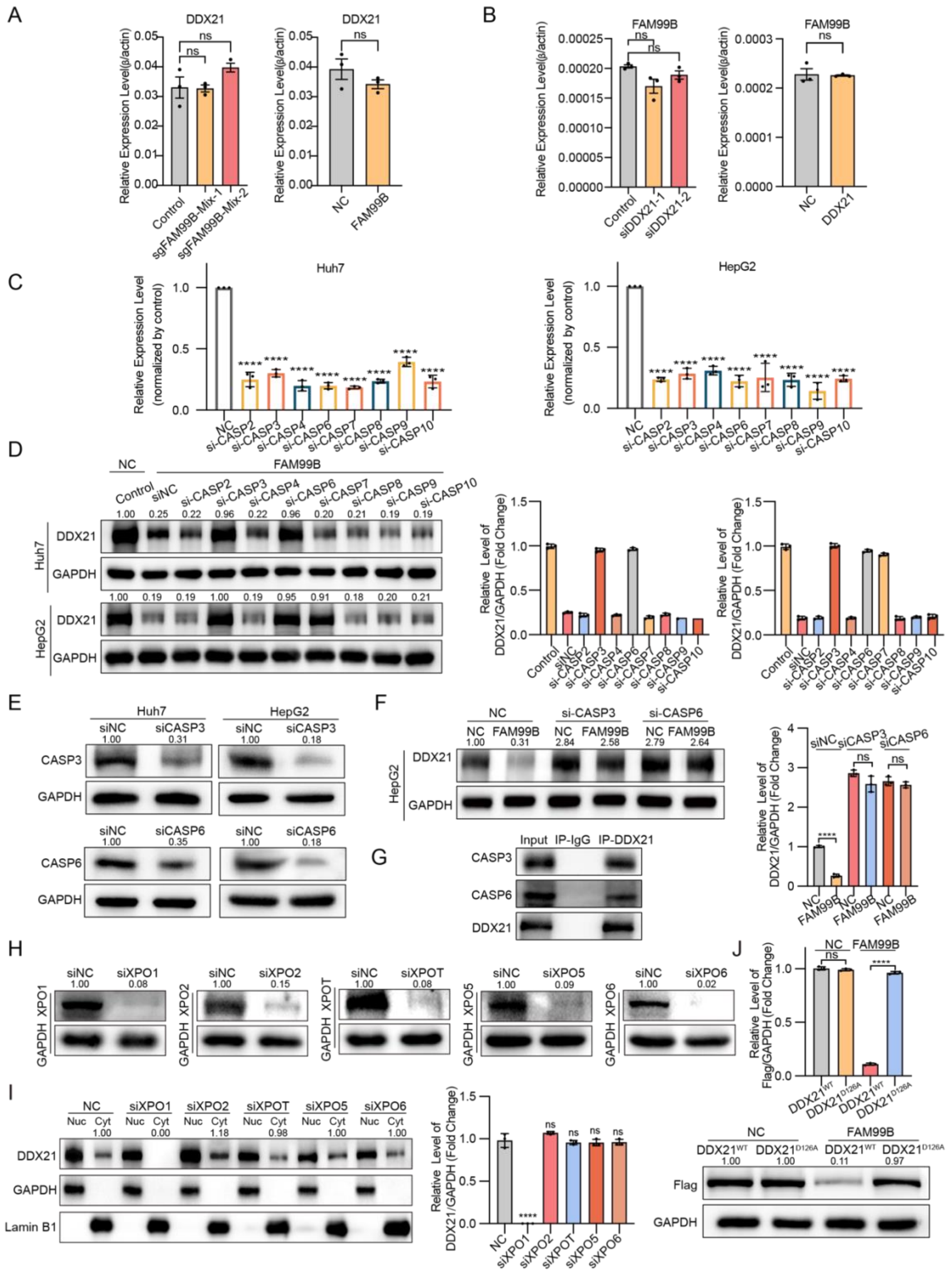

Figure S4. FAM99B decreases the DDX21 protein level via casp3/6.

A. Relative mRNA level of DDX21 in Huh7 cells with FAM99B overexpression or knockout, as determined via qPCR. Data are represented as the mean ± SD (n = 3). One-way ANOVA with correction for multiple comparisons. **B.** Relative RNA level of FAM99B in Huh7 cells with DDX21 overexpression or knockdown, as determined via qPCR. Data are represented as the mean ± SD (n = 3). One-way ANOVA with correction for multiple comparisons. **C.** We assessed the efficiency of knocking down caspase2, caspase3, caspase4, caspase6, caspase7, caspase8, caspase9, and caspase10 in Huh7 and HepG2 cells using qPCR. Normalize the expression level of NC group caspasases to 1, and calculate the expression level of caspase 2, caspase 3, caspase 4, caspase 6, caspase 7, caspase 8, caspase 9, caspase 10 in Huh7 and HepG2 cells after being knocked down. Data are represented as the mean ± SD (n = 3). One-way ANOVA with correction for multiple comparisons. **D.** The protein expression level of DDX21 in Huh7 (Left) and HepG2 (Right) cells with stable overexpression of FAM99B after knockdown of CASP2, CASP3, CASP4, CASP6, CASP7, CASP8, CASP9 or CASP10 was measured via Western blotting, followed by statistical analysis. Data are represented as the mean ± SD (n = 3). One-way ANOVA with correction for multiple comparisons. **E.** The knockdown efficiency of CASP3, CASP6 and CASP7 was determined by Western blotting. **F.** The protein expression level of DDX21 after knockdown of CASP3 and CASP6 in HepG2 cells with stable overexpression of FAM99B was measured by Western blotting, followed by statistical analysis. Data are represented as the mean ± SD (n = 3). Unpaired t test. **G.** Immunoprecipitation was

used to detect the association between caspase3/6 and DDX21 after FAM99B overexpression in Huh7 cells, followed by statistical analysis. H. Western blot analysis of the knockdown efficiency of XPO1, XPO2, XPOT, XPO5, and XPO6 in Huh7 cells. **I.** Western blot analysis of the changes in DDX21 protein levels in the nucleus and cytoplasm of FAM99B-overexpressing Huh7 cells after knockdown of XPO1, XPO2, XPOT, XPO5, and XPO6, followed by statistical analysis. Data are represented as the mean ± SD (n = 3). One-way ANOVA with correction for multiple comparisons. **J.** Huh7 cells stably overexpressing FAM99B were transfected with Flag-tagged $DDX21^{WT}$ or $DDX21^{D126A}$, and 24 h after transfection, Western blot analysis was performed, followed by statistical analysis.ALL data are represented as the mean ± SD (n = 3). One-way ANOVA with correction for multiple comparisons.

Supplementary Figure S5.

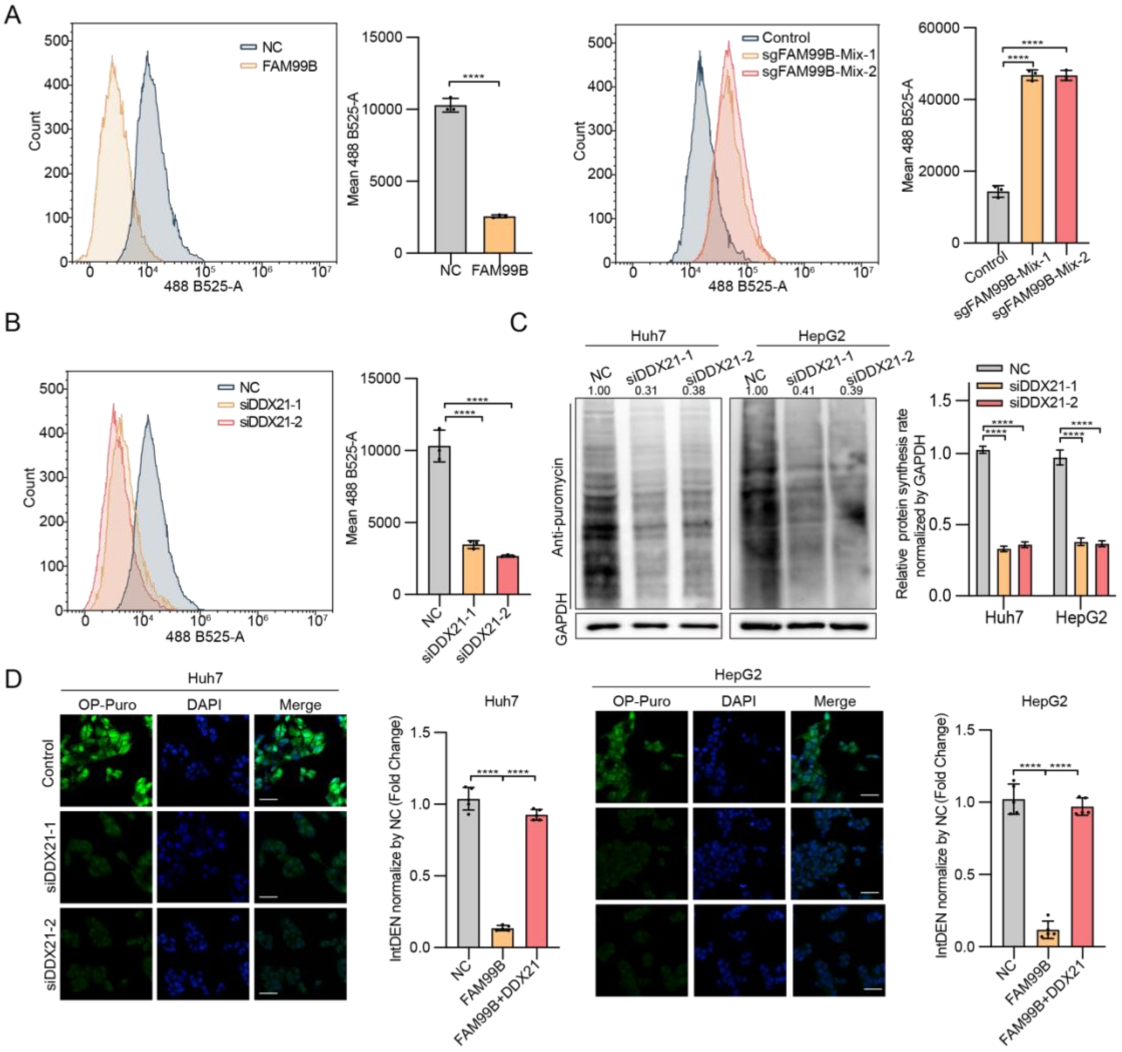

Figure S5. Knockdown of DDX21 inhibits protein synthesis in HCC.

A. FASC analysis was used to detect the protein synthesis rate in Huh7 cells after FAM99B overexpression or knockout, followed by statistical analysis. Data are represented as the mean ± SD (n = 3). Unpaired t test or One-way ANOVA with correction for multiple comparisons. **B.** FASC analysis was used to detect the protein synthesis rate in Huh7 cells after DDX21 knockdown, followed by statistical analysis. Data are represented as the mean ± SD (n = 3). One-way ANOVA with correction for

multiple comparisons. **C.** Effects of DDX21 knockdown on protein synthesis in Huh7 and HepG2 cells, as determined by the SUnSET method, followed by statistical analysis. Data are represented as the mean ± SD (n = 3). One-way ANOVA with correction for multiple comparisons. **D.** The effects of DDX21 knockdown on protein synthesis in Huh7 and HepG2 cells were also evaluated via an OP-Puro assay. Randomly select 5 fields of view, calculate the fluorescence Integrated Density (IntDen) using ImageJ, and perform statistical analysis. Data are represented as the mean ± SD (n = 5). One-way ANOVA with correction for multiple comparisons. Scale bar, 25 μm.

Supplementary Figure S6.

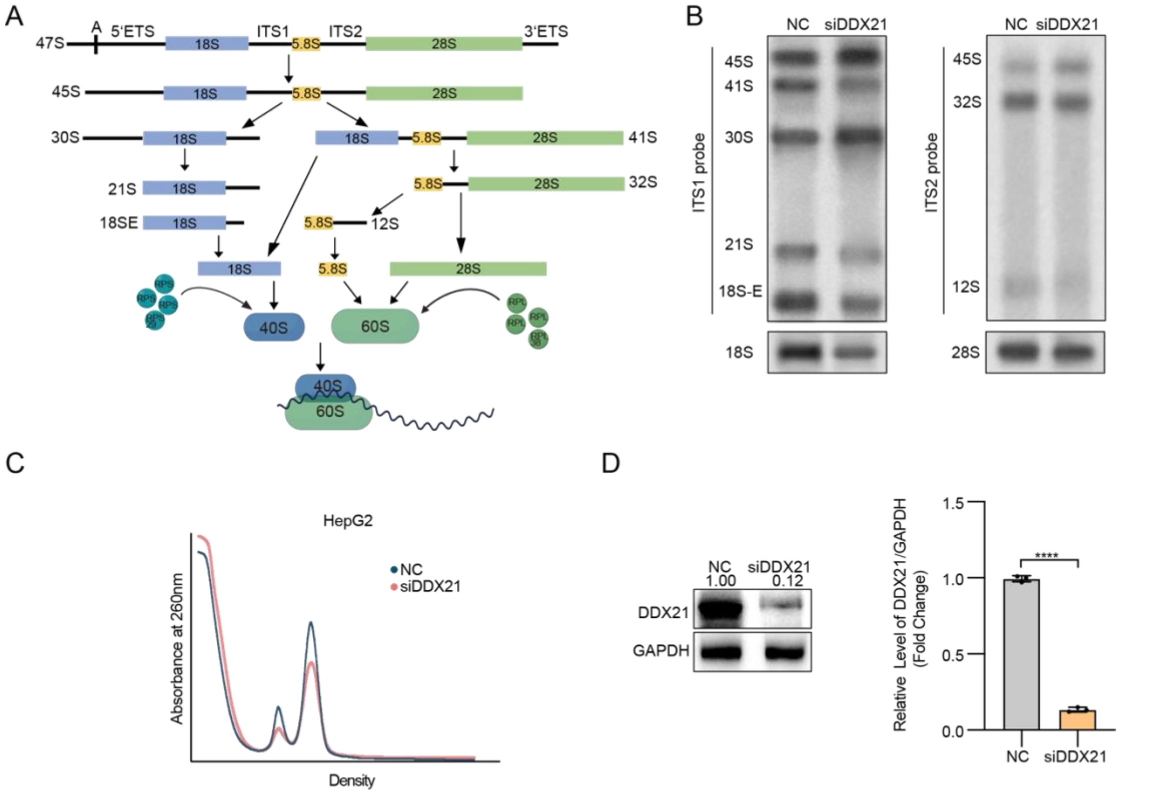

Figure S6. DDX21 regulates rRNA processing and ribosome biosynthesis in HCC.

A. Diagram of the Ribosome Biogenesis Process. Ribosome biogenesis begins with the transcription of ribosomal DNA into 47S pre-rRNA by RNA polymerase I and a series of regulatory factors. Subsequently, the 47S pre-rRNA undergoes cleavage, removal of excess intergenic sequences, and multiple modifications to produce mature 18S, 5.8S, and 28S rRNAs. These rRNAs associate with various ribosomal proteins to form pre-40S and pre-60S particles. Finally, the 40S and 60S ribosomal subunits are assembled into mature ribosomes in the cytoplasm. The 47S pre-rRNA is processed and categorized as follows: 5′ external transcribed spacer (5′ ETS), mature 18S rRNA, internal transcribed spacer 1 (ITS1), mature 5.8S rRNA, internal transcribed spacer 2 (ITS2), mature 28S rRNA, and 3′ external transcribed spacer (3′ ETS). The A site is the cleavage site in pre-rRNA processing. **B.** ITS1, ITS2, 18S and 28S probes were used to determine the expression levels of pre-rRNAs and mature rRNAs after DDX21

knockdown in Huh7 cells were analyzed by Northern blotting (n=3 independent experiments). **C.** Ribosomal profiles after puromycin-mediated dissociation following the knockdown of DDX21. **D.** The protein expression level of DDX21 after knockdown of DDX21 was measured by Western blotting, followed by statistical analysis. Data are represented as the mean ± SD (n = 3). Unpaired t test.

1 **Supplementary Figure S7.**

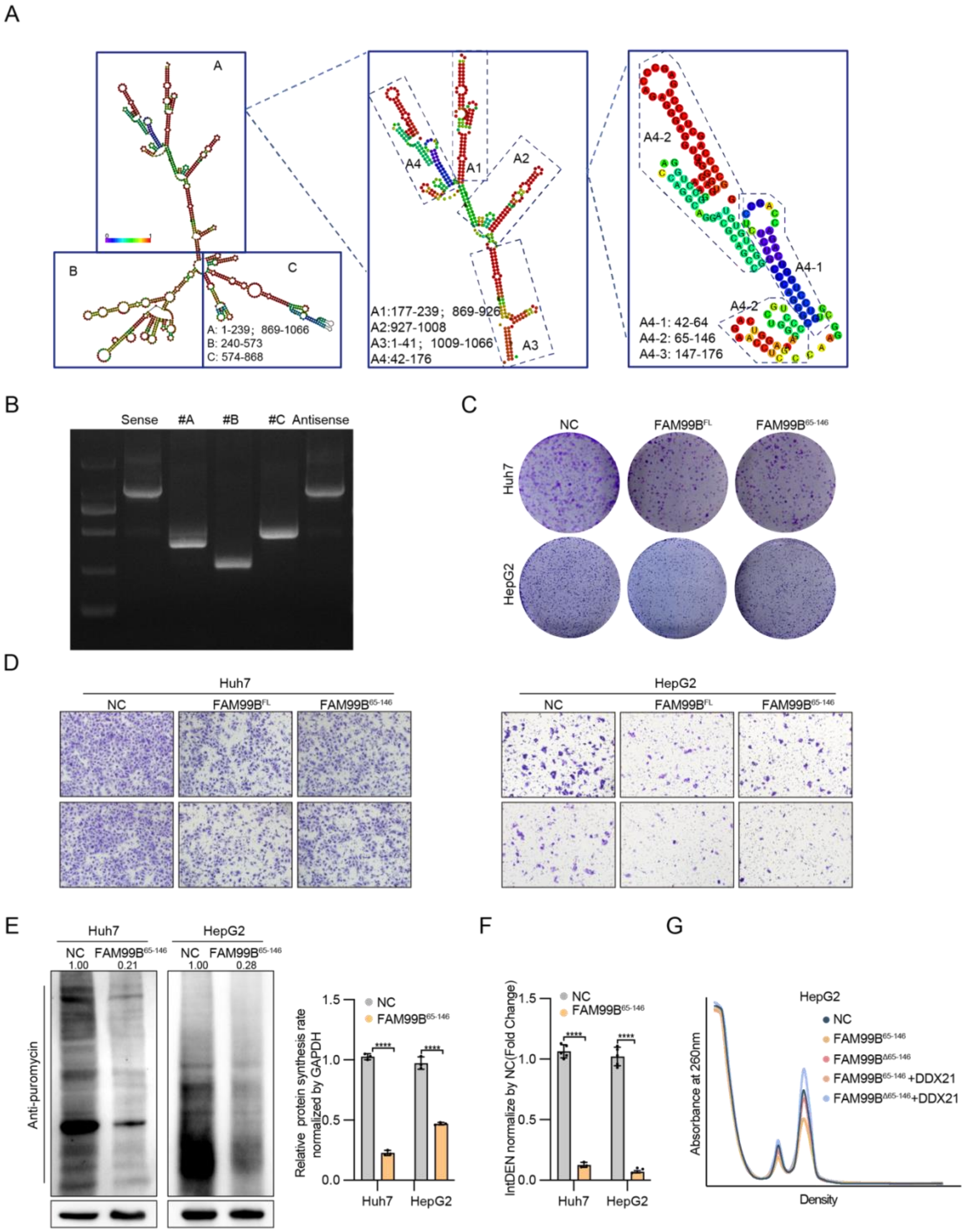

2

3 **Figure S7. The FAM99B$^{65\text{-}146}$ fragment plays a tumor-suppressive role in HCC**

4 **cells.**

A. Secondary structure of FAM99B as determined via the RNAfold web server (http://rna.tbi.univie.ac.at/cgibin/RNAWebSuite/RNAfold.cgi). **B.** Detection of FAM99B truncations A, B and C by DNA electrophoresis. **C.** Colony formation assays of HCC cells overexpressing $FAM99B^{65-146}$. **D.** Migration and invasion assays of Huh7 and HepG2 cells overexpressing $FAM99B^{65-146}$. E. The effects of $FAM99B^{65-146}$ overexpression on protein synthesis in Huh7 and HepG2 cells were evaluated by the SUnSET method, followed by statistical analysis. Data are represented as the mean ± SD (n = 3). Unpaired t test. **F.** After overexpression of $FAM99B^{65-146}$ in Huh7 and HepG2 cells, the changes in fluorescence Integrated Density in the OP-Puro assay were statistically analyzed. Data are represented as the mean ± SD (n = 5). Unpaired t test. **G.** Ribosomal subunit profiles after puromycin-mediated dissociation conditions in Huh7 cells: overexpression of $FAM99B^{65-146}$, overexpression of $FAM99B^{\Delta 65-146,}$ co-expression of $FAM99B^{65-146}$ and DDX21, and co-expression of $FAM99B^{\Delta 65-146}$ and DDX21 (n=3 independent experiments). *p < 0.05, **p < 0.01, ***p < 0.001, ****p < 0.0001, and ns, not significant.

Supplementary Figure S8.

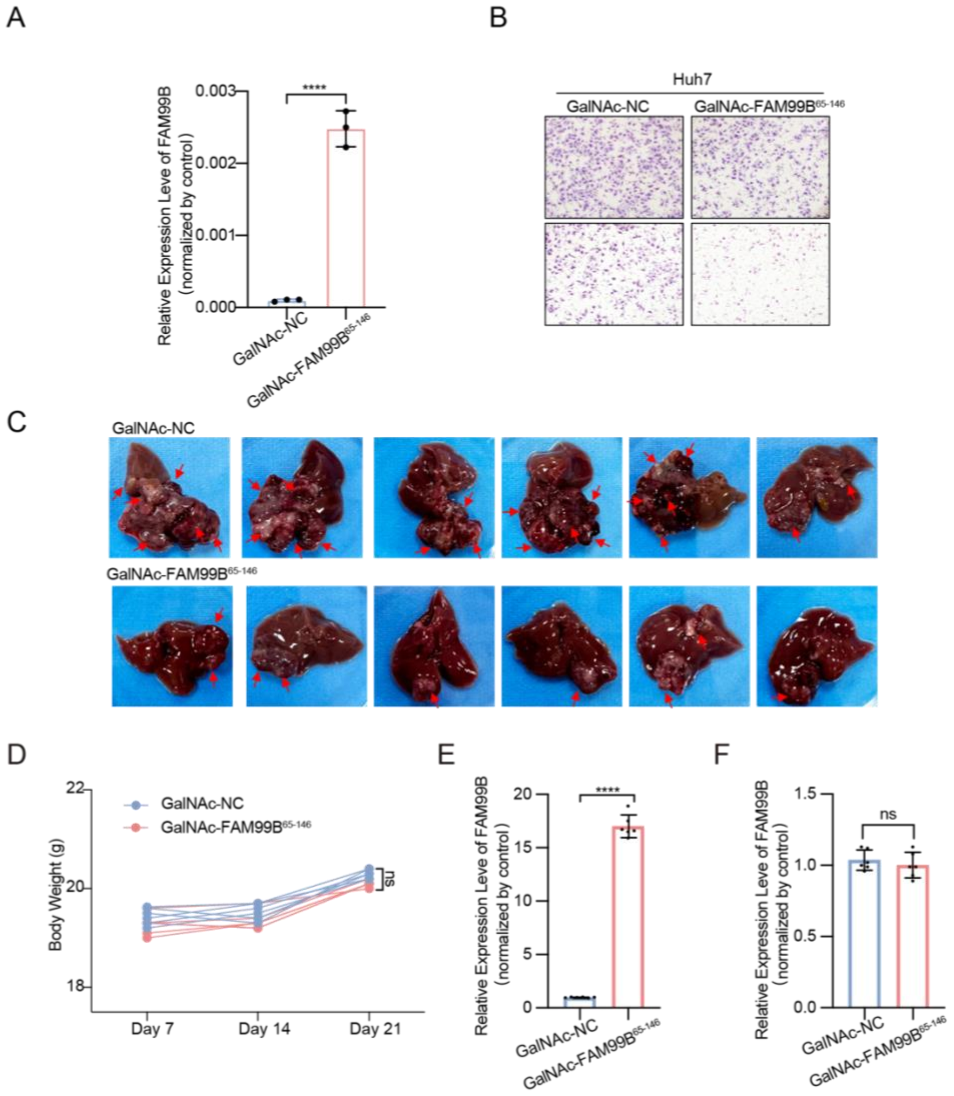

Figure S8. GalNAc-FAM99B$^{65\text{-}146}$ inhibits the growth and metastasis of orthotopic liver xenografts in nude mice.

A. The overexpression efficiency of FAM99B$^{65\text{-}146}$ in Huh7 cells was determined via qPCR. Data are represented as the mean ± SD (n = 3). Unpaired t test. **B.** Migration and invasion assays of Huh7 cells overexpressing FAM99B$^{65\text{-}146}$. **C.** Growth of orthotopic liver tumors in the GalNAc-NC and GalNAc-FAM99B$^{65\text{-}146}$ treatment groups (n = 6). **D.** Body weight changes of mice in the control group (GalNAc-NC) and treatment

group (GalNAc-FAM99B$^{65-146}$) from day 7 to day 21(n = 6). Two-way ANOVA with correction for multiple comparisons. **(E, F)** After euthanizing the mice on day 21, qPCR analysis was performed to measure FAM99B$^{65-146}$ expression in the liver (e) and lung (F) tissues from the GalNAc-NC and GalNAc-FAM99B$^{65-146}$ treatment groups. Data are represented as the mean ± SD (n = 3). Unpaired t test. *$p < 0.05$, **$p < 0.01$, ***$p < 0.001$, ****$p < 0.0001$.
